# Multi-Parametric Molecular Imaging of the Brain Using Optimized Multi-TE Subspace MRSI

**DOI:** 10.1109/TBME.2023.3349375

**Published:** 2024-05-20

**Authors:** Zepeng Wang, Yahang Li, Chang Cao, Aaron Anderson, Graham Huesmann, Fan Lam

**Affiliations:** Department of Bioengineering, University of Illinois Urbana-Champaign, USA.; Department of Bioengineering, University of Illinois Urbana-Champaign, USA.; Beckman Institute for Advanced Science and Technology, University of Illinois Urbana-Champaign, USA.; Beckman Institute for Advanced Science and Technology, University of Illinois Urbana-Champaign, USA.; School of Molecular and Cellular Biology, University of Illinois Urbana-Champaign, USA.; Department of Bioengineering, Beckman Institute for Advanced Science and Technology, University of Illinois Urbana-Champaign, Urbana, IL 61801 USA

**Keywords:** Multi-TE MR spectroscopic imaging (MRSI), multi-parametric imaging, subspace imaging, experimental optimization

## Abstract

**Objective:**

To develop a novel multi-TE MR spectroscopic imaging (MRSI) approach to enable label-free, simultaneous, high-resolution mapping of several molecules and their biophysical parameters in the brain.

**Methods:**

The proposed method uniquely integrated an augmented molecular-component-specific subspace model for multi-TE ^1^H-MRSI signals, an estimation-theoretic experiment optimization (nonuniform TE selection) for molecule separation and parameter estimation, a physics-driven subspace learning strategy for spatiospectral reconstruction and molecular quantification, and a new accelerated multi-TE MRSI acquisition for generating high-resolution data in clinically relevant times. Numerical studies, phantom and in vivo experiments were conducted to validate the optimized experiment design and demonstrate the imaging capability offered by the proposed method.

**Results:**

The proposed TE optimization improved estimation of metabolites, neurotransmitters and their $T_{2}’s$ over conventional TE choices, e.g., reducing variances of neurotransmitter concentration by ~ 40% and metabolite $T_{2}$ by ~ 60%. Simultaneous metabolite and neurotransmitter mapping of the brain can be achieved at a nominal resolution of 3.4 × 3.4 × 6.4 mm^3^. High-resolution, 3D metabolite $T_{2}$ mapping was made possible for the first time. The translational potential of the proposed method was demonstrated by mapping biochemical abnormality in a post-traumatic epilepsy (PTE) patient.

**Conclusion:**

The feasibility for high-resolution mapping of metabolites/neurotransmitters and metabolite $T_{2}’s$ within clinically relevant time was demonstrated. We expect our method to offer richer information for revealing and understanding metabolic alterations in neurological diseases.

**Significance:**

A novel multi-TE MRSI approach was presented that enhanced the technological capability of multi-parametric molecular imaging of the brain. The proposed method presents new technology development and application opportunities for providing richer molecular level information to uncover and comprehend metabolic changes relevant in various neurological applications.

## I. Introduction

Magnetic resonance spectroscopic imaging (MRSI) is the only in vivo molecular imaging modality that can achieve simultaneous mapping of major metabolites such as N-acetylaspartate (NAA), creatine (Cr), choline (Cho), myoinositol (mI) and neurotransmitters such as glutamate (Glu) and γ-aminobutyric acid (GABA) [1]. Such capability allows for investigating metabolic alterations that are inaccessible by anatomical and functional scans in various neurological diseases, e.g., brain cancer [2], [3], stroke [4], [5], epilepsy [6], and neurodegenerative disorders [7]. Beyond the abundance of different molecules, MRSI also affords estimation of biophysical parameters of various metabolites, such as $T_{1}$ and $T_{2}$ relaxation constants. Such multiparametric information can not only help improve metabolite quantification [8], [9] but also provide potentially a richer set of biomarkers for a range of diseases [10], [11].

J-resolved MRSI is a common choice to achieve multiparametric molecular imaging, by adding a second spectral/TE dimension (a.k.a. the indirect frequency) to encode the J-coupling evolutions and $T_{2}$ decays of different molecules. While the additional encoding dimension allows better molecule separation, J-resolved MRSI poses significant technical challenges: (1) the inherently low SNR of MRSI, and (2) higher dimensionality due to the need of acquiring data at multiple TEs, which further limits the trade-off between SNR, resolution and imaging speed. Therefore, practical J-resolved MRSI experiments are mostly limited to either single-voxel [10] or low-resolution imaging acquisitions with large voxel sizes (~1 cm^3^) [12], [13]. Furthermore, it has been consistently demonstrated that the conventional uniform-TE sampling for visualizing 2D spectra is a sub-optimal choice for parameter estimation [14]. Significant efforts have been made on accelerating J-resolved MRSI, including fast spatiospectral encoding sequences such as echo-planar spectroscopic imaging (EPSI) [12] and spiral CSI [15], [16], model-based reconstruction from sparsely-sampled noisy measurements [17], [18], low-rank tensor modeling (which requires many TEs) [19], [20], [21], [22] to realize acceleration, and non-uniform TE sampling for optimized acquisition efficiency for the estimation of a specific set of molecular parameters [23]. However, the combinations of speed, resolution, SNR and organ coverage achieved are still highly limited. Recently, subspace imaging methods based on the SPICE framework have been presented for accelerating J-resolved MRSI of the brain by exploring the spatial-temporal-TE correlations within the high-dimensional data [24]. Nevertheless, brain coverage remains limited and the ability to simultaneously map metabolites, neurotransmitters as well as their relaxation parameters has never been demonstrated.

We presented here an augmented subspace imaging approach and demonstrate the feasibility of fast, high-resolution, simultaneous multi-parametric molecular imaging of the brain (molecule concentrations and their relaxation parameters). Specifically, we described an augmented multi-TE subspace model to represent the high-dimensional J-resolved/multi-TE MRSI data for spatiospectral reconstruction and quantification. Under this model, we proposed an estimation-theoretic analysis that allowed task-specific optimal experiment designs for separation of spectrally overlapping metabolites and neurotransmitters (Glu and GABA) and metabolite $T_{2}$ mapping. A physics-driven, data-adaptive multi-TE subspace learning strategy was proposed to pre-learn the molecule-component-specific subspaces for experimental optimization, high-SNR spatiospectral reconstruction and a multi-step, multi-parameter quantification.

To realize the proposed method, a new fast sequence synergizing adiabatic refocusing, rapid spatiotemporal encoding, (k, t, TE)-space sparse sampling and interleaved high-resolution water navigators was designed and implemented on a 3 T system. The proposed acquisition can produce high-resolution, multi-TE MRSI data with optimized TE selection in clinically relevant times.

Numerical simulations and phantom experiments have been conducted to validate the proposed experimental design and quantification strategies. In vivo multi-TE MRSI experiments demonstrated the feasibility of both high-resolution metabolite/neurotransmitter mapping (e.g., an optimized 2-TE scan in ~15 mins) and metabolite $T_{2}$ mapping (e.g., an optimized 4-TE scan in ~25 mins) with a nominal 3.4 × 3.4 × 6.4 mm^3^ resolution. The exciting capability of simultaneously mapping metabolite and neurotransmitter was also evaluated on a PTE patient, which further demonstrated the clinical potential of the proposed method. The following sections describe the proposed method in details.

## II. Proposed Method

### A. An Augmented Subspace Model for Multi-TE MRSI

The proposed method is founded on the following augmented subspace model for the desired multi-dimensional function of interest, ρ(r,t,TE), in J-resolved MRSI (assuming nuisance water and subcutaneous lipids removed) [24]:

(1) $$\rho (r,t,\text{TE})=\sum_{m=1}^{M}\sum_{l_{m}=1}^{L_{m}}c_{l_{m}}(r)v_{l_{m}}(t,\text{TE}),$$

where t and TE denote the chemical shift (or free induction decay, FID) and J-evolution (or TE) dimensions, respectively, m indexes signal components which can be defined differently for specific applications. This model exploits the assumption that each signal component (e.g., metabolites vs. neurotransmitters) resides in its own multi-TE subspace, spanned by $\left\{v_{l_{m}}(t,\text{TE})\right\}$, enabling their further separation exploiting both chemical shift and J-coupling differences. $L_{m}$ is the model order for each component and $c_{l_{m}}$ are the component-specific spatial coefficients. This model further reduces the dimensionality for the J-resolved MRSI data than treating each TE independently. The dimensionality of each multi-TE subspace ($L_{m}$) is typically lower than the sum of dimensions for individual-TE subspaces, by exploiting correlations between t and TE [24]. This augmented subspace model will serve as the framework for spatiospectral reconstruction, optimal multi-TE experiment design and multi-parametric estimation, which can be task-specific, e.g., for either optimized metabolite and neurotransmitter signal separation or optimized molecule-specific $T_{2}$ estimation or both.

### B. Physics-Driven Subspace Learning

A key issue for the proposed model is the learning of component-specific subspace/basis, $v_{l_{m}}(t,\text{TE})$. The connections between FIDs generated by a biophysical quantification model residing on a manifold and low-dimensional subspace approximations have been established [25], [26], [27]. This motivated a physics-driven approach for learning $v_{l_{m}}(t,\text{TE})$. Specifically, we chose a well-established multi-TE spectral fitting model here

(2) $$s(n,\text{TE})=e^{i\varphi_{\text{TE}}}\sum_{m=1}^{M}c_{m}\phi_{m,\text{TE}}(n\Delta t)e^{-\left[\text{TE}+n\Delta t\right]∕T_{2,m}}\;\;e^{-\left[n\Delta t\right]∕T_{2,m}^{\prime }}e^{-i2\pi \Delta f_{m}n\Delta t}e^{-{\left[n\Delta t\right]}^{2}g_{\text{TE}}},$$

where s(n,TE) represents the multi-TE FIDs (after spatiospectral reconstruction), Δt denotes the temporal sampling rate with n the sampling index, $\phi_{m,\text{TE}}$ is the TE-dependent molecule basis that can be predicted by quantum mechanical (QM) simulations for a specific excitation scheme used in data acquisition. The variables $\left\{c_{m}\right\}$, $\left\{T_{2,m}\right\}$, $\left\{T_{2,m}^{\prime }\right\}$ and $\left\{\Delta f_{m}\right\}$ denote the concentrations, relaxation parameters, additional decay rates (due to intravoxel field inhomogeneity) and frequency displacements, respectively, $\varphi_{\text{TE}}$ is a TE-dependent zeroth-order phase, and $g_{\text{TE}}$ is an additional TE-dependent Gaussian lineshape distortion factor.

**Fig. 1.**
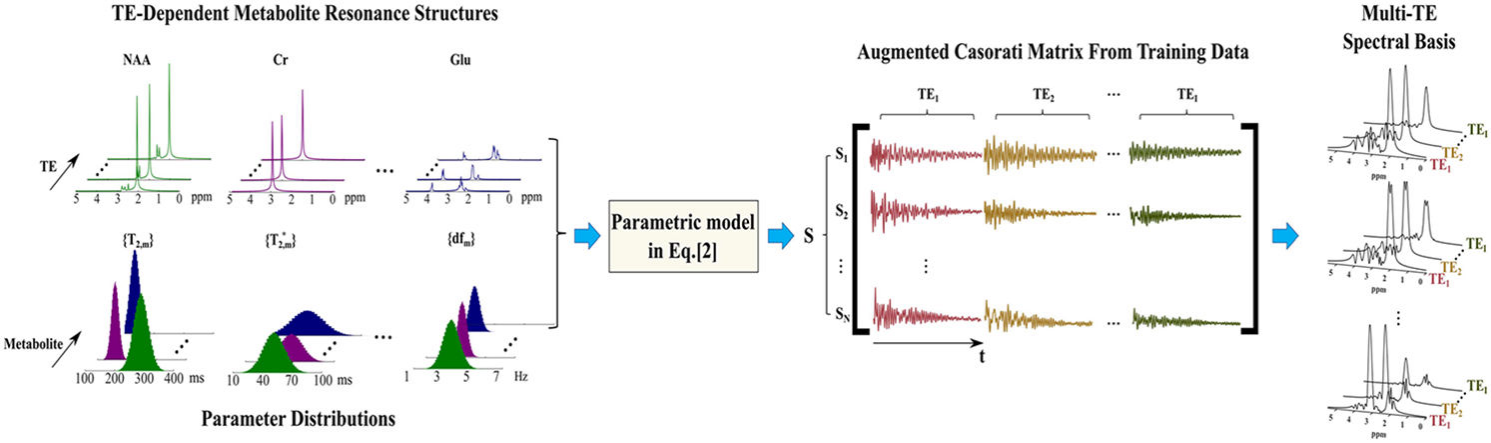
Physics-driven multi-TE subspace learning: TE-dependent metabolite resonance structures were obtained from QM simulations (left column, top). Samples of spectral parameters were drawn from distributions estimated from literature values and/or experimental data (left column, bottom). These parameters were fed into the biophysical model (2) to synthesize a large number of multi-TE training FIDs (S in the middle column), which subsequently formed an augmented Casorati matrix. Specifically, training FIDs (denoted as $\left\{S_{n}\right\}$) were stacked along the row direction, and for each sample, TE-dependent FIDs were concatenated along the column direction. A set of multi-TE spectral basis can then be estimated by SVD (most right column, basis shown after Fourier transform and reorganized along the TE dimension).

With $\phi_{m,\text{TE}}$ from QM simulations, Eq. (2) allows us to generate realistic FIDs/spectra via sampling the space of $\left\{c_{m}\right\}$, $\left\{T_{2,m}\right\}$, $\left\{T_{2,m}^{\prime }\right\}$, $\left\{\Delta f_{m}\right\}$, $\left\{\varphi_{\text{TE}}\right\}$, $\left\{g_{\text{TE}}\right\}$. This can be done by drawing random samples from assumed/estimated distributions of these parameters, from literature values or experiment data [28], e.g., $T_{2}$ ranges reported in Ref. [29]. For parameters without good prior distributions, uniformly random distributions can be assumed, e.g., −π to π for the phase terms. These sampled spectral parameters can be fed into Eq. (2) to generate a large set of multi-TE FIDs (${\left\{s_{i}(n,\text{TE})\right\}}_{i=1}^{N}$) for specific molecular components (training samples). An augmented Casorati matrix can then be formed from these samples, e.g., with each multi-TE FIDs being a row and FIDs concatenated along TE. Note that these matrices can be constructed for individual molecules or their combinations, from which component-specific subspaces can be extracted, e.g., using SVD. This physics-driven subspace learning is illustrated in Fig. 1.

### C. Estimation-Theoretic Optimization of J-Resolved MRSI Acquisitions

It has been shown that an optimized set of arbitrarily spaced TEs instead of the conventional uniform-TE sampling can improve the quantification of specific molecules [14], [23], [30]. Our model in Eq. (1) with learned subspaces provides a new avenue to optimize J-resolved experiments. Here, we performed estimation-theoretic TE optimization for (1) metabolite and neurotransmitter separation and (2) molecule $T_{2}$ estimation, respectively.

#### 1) Experimental Optimization for Metabolite and Neurotransmitter Separation:

To optimize the separation of metabolite and neurotransmitter signals, we can adapt Eq. (1) as follows:

(3) $$\rho (r,t,\text{TE})=\sum_{l_{met}=1}^{L_{met}}c_{l_{met}}(r)v_{l_{met}}(t,\text{TE})\;\;+\sum_{l_{glx}=1}^{L_{glx}}c_{l_{glx}}(r)v_{l_{glx}}(t,\text{TE})\;\;+\sum_{l_{gaba}=1}^{L_{gaba}}c_{l_{gaba}}(r)v_{l_{gaba}}(t,\text{TE}).$$

The overall signal is decomposed into a “major” metabolite component (e.g., including NAA, Cr, Cho, mI and Taurine (Tau)), a Glx component (glutamate and glutamine) and a GABA component, spanned by pre-learned $\left\{v_{l_{met}}(t,\text{TE})\right\}$, $\left\{v_{l_{glx}}(t,\text{TE})\right\}$, and $\left\{v_{l_{gaba}}(t,\text{TE})\right\}$, respectively. $L_{x}$ denotes the model order for individual components. Compared to the single-molecule subspaces for spectral quantification in Ref. [31], this model allows further reduction of dimensionality and enables TE optimization specific to the task of separating major metabolites, Glx and GABA.

Specifically, after discretization and considering measurement noise, Eq. (3) can be rewritten as:

(4) $$\rho =[V_{met},V_{glx},V_{gaba}]\left[\begin{array}{c} c_{met}\\ c_{glx}\\ c_{gaba} \end{array}\right]+n,$$

where ρ is the matrix representation of ρ(r,t,TE) defined over a set of voxels and time points for a given spatiospectral resolution (with the t dimension concatenated across TEs), and n captures the white Gaussian measurement noise with a standard deviation δ. $V_{x}’s$ are matrix representations of the component-specific multi-TE subspaces in Eq. (3), and $c_{x}’s$ are vectors containing respective spatial coefficients. This linear representation affords an efficient estimation-theoretic analysis. Denoting $\hat{c}={\left[{\hat{c}}_{met}^{T},{\hat{c}}_{glx}^{T},{\hat{c}}_{gaba}^{T}\right]}^{T}$ as coefficient estimates, the Cramer-Rao lower bound (CRLB) for $\hat{c}$ can be derived as:

(5) $$COV(\hat{c})\geq \delta^{2}{\left(V^{H}V\right)}^{-1}\;\;=\delta^{2}{\left[\begin{array}{ccc} V_{\text{met}}^{H}V_{\text{met}} & V_{\text{met}}^{H}V_{\text{glx}} & V_{\text{met}}^{H}V_{\text{gaba}}\\ V_{\text{glx}}^{H}V_{\text{met}} & V_{\text{glx}}^{H}V_{\text{glx}} & V_{\text{glx}}^{H}V_{\text{gaba}}\\ V_{\text{gaba}}^{H}V_{\text{met}} & V_{\text{gaba}}^{H}V_{glx} & V_{\text{gaba}}^{H}V_{\text{gaba}} \end{array}\right]}^{-1}$$

where $\delta^{2}{\left(V^{H}V\right)}^{-1}$ is the inverse Fisher Information Matrix (iFIM). The CRLB for individual components can then be obtained by summing up the diagonal elements in the iFIM corresponding to metabolite, Glx or GABA basis. As all the $V_{x}’s$ are TE-depend, the CRLBs depend on TE selection. Therefore, we can choose the optimal TE combinations to minimize the CRLBs for different components, e.g., Glx, GABA or both. More specifically, we devised a greedy algorithm to gradually add TE to selected subset until the CRLB stopped decreasing, with an equivalent scan time constraint (e.g., 2 TEs with 2 averages vs. 4 TEs with 1 average). We observed similar TEs predicted this way compared to those from an exhaustive search (Supporting Information Fig. S1). In particular, using learned subspaces that are specific to our proposed pulse sequence (described below), an optimal 2-TE combination of 65 and 80 ms was determined for minimizing the estimation variances of GABA, which is similar to the TEs obtained previously using more standard parametric models [23]. It is also worth noting that based on Eq. (5), the TE optimization is essentially searching for TEs that minimize the overlap between different signal subspaces.

#### 2) Experimental Optimization for Metabolite $T_{2}$ Estimation:

For metabolite $T_{2}$ estimation, we performed estimation-theoretic analysis using the multi-TE parametric model in Eq. (2). More specifically, the Fisher Information Matrix (FIM) can be derived by taking the expectation of the derivatives of the log-likelihood function, similarly done in previous CRLB analysis for single/multi-TE quantification (see Appendix for details) [14].

**Fig. 2.**
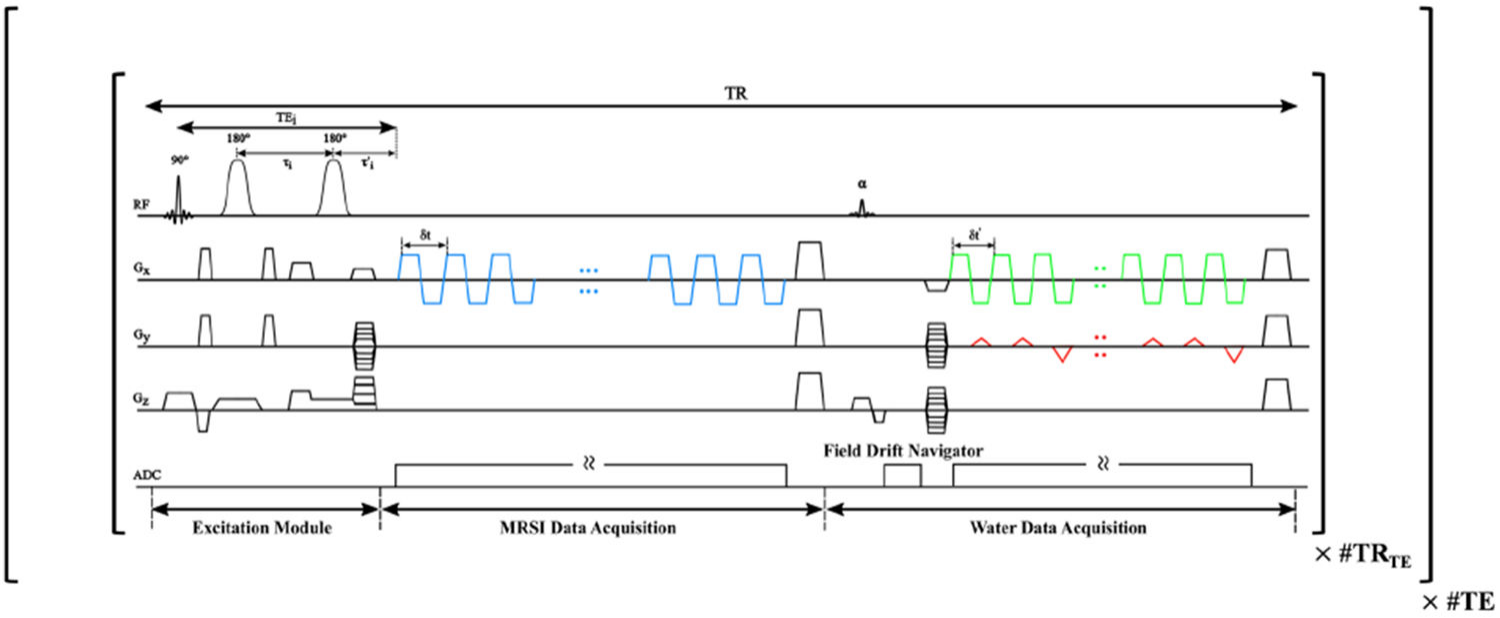
Proposed sequence: The excitation uses a spatially selective excitation pulse and a pair of adiabatic refocusing pulses. Different TEs are realized by adjusting the delay between the adiabatic pulses ($\tau_{i}$). The MRSI acquisition module uses an EPSI trajectory (starting at TE, $\tau_{i}^{\prime }$) for fast spatiospectral encoding with phase encoding along y and z. Echospacing is δt. A set of field drift navigator (readout without phase encoding) and spatiotemporally encoded water imaging data are acquired after a first spoiler and a small-angle excitation ($\alpha =10^{o}$). These modules are repeated until the desired MRSI resolution is achieved at each TE.

With the FIM derived, the TE-number and value-dependent CRLBs for $\left\{T_{2,m}\right\}$ were calculated by summing up the corresponding indices of *targeted* parameters from the diagonal elements in the iFIM. In this work, we chose the TE combination that minimizes the $T_{2}$ estimation CRLBs for NAA, Cr and Cho (while this can be done for other molecules as well). An equivalent-time comparison was used as in the metabolite/neurotransmitter separation case (i.e., any TE can be acquired multiple times). We performed the CRLB calculation for 2 to 12 TEs, using an exhaustive search for the first 2 optimal TEs followed by a greedy search for additional TEs. This allowed us to identify an optimized 4-TE combination (Supporting Information Fig. S2), i.e., 35, 200, 245, and 275 ms, minimizing the overall CRLB for NAA, Cr and Cho $T_{2}$ estimation. As shown by the Monte-Carlo simulation results in Fig. 2 (right panel), this combination led to clearly improved $T_{2}$ estimation over TE choices used in previous literature [12].

### D. Multi-Parametric Quantification

As spatiospectral reconstruction using learned subspaces has been extensively described [24], [32], [33], we focus here on our quantification strategy to extract metabolites, neurotransmitters and their $T_{2}$ parameters, from the spatio-temporal-TE reconstruction, $\hat{\rho }(r,t,\text{TE})$. The key challenges here are (1) the large dynamic range for signals from different molecules (major metabolites such as NAA and Cr much stronger than Glx and GABA) and (2) directly fitting $T_{2}$ using the complicated nonlinear parametric model [34], [35] voxel-wise yields large estimation variance. Inspired by the subspace-based approach [31], we proposed here an enhanced union-of-subspaces (UoSS)-based, multi-step strategy integrating parametric and subspace fitting to address these issues, which offers flexible task-specific estimation.

First, we conducted a parametric fitting of $\hat{\rho }(r,t,\text{TE})$ using Eq. (2) by a VARPRO [36] algorithm, which provided a set of initially separated molecules of interest, from which component-specific subspaces were extracted (using a similar approach described in subspace learning section). Second, using these subspaces, a UoSS-based fitting was performed to the original $\hat{\rho }(r,t,\text{TE})$ for an initial separation of a metabolite component (including NAA, Cr, Cho, mI and Tau) and a neurotransmitter component (Glx and GABA). Specifically, we represented $\hat{\rho }(r,t,\text{TE})$ as

(6) $$\hat{\rho }(r,t,\text{TE})=\sum_{l_{met}=1}^{L_{met}}c_{l_{met}}(r){\hat{v}}_{l_{met}}(t,\text{TE})\;\;+\sum_{l_{nt}=1}^{L_{nt}}c_{l_{nt}}(r){\hat{v}}_{l_{nt}}(t,\text{TE}),$$

where $\left\{{\hat{v}}_{l_{met}}(t,\text{TE})\right\}$ and $\left\{{\hat{v}}_{l_{nt}}(t,\text{TE})\right\}$ were the multi-TE augmented metabolite subspace from the first parametric fitting and learned neurotransmitter subspace. Note that this step better compensates the lineshape distortions not completely captured by voxel-wise parametric fitting and allows the incorporation of spatial regularization. The separated metabolite component will be referred to as ${\hat{\rho }}_{met}(r,t,\text{TE})$ below.

Third, a *targeted* UoSS fitting was applied to ${\hat{\rho }}_{met}(r,t,\text{TE})$ to estimate the spatial distributions of individual metabolites. More specifically, we formulated the problem as

(7) {clq∗(r)}=argmin{clq}‖ρ^met(r,t,TE)‖−‖∑q=1Q∑lq=1Lqclq(r)v^lq(t,TE)‖22+∑q=1QλqRq({clq(r)}),

where $\left\{c_{l_{q}}^{*}\right\}$ are the estimated spatial coefficients for different metabolites indexed by q, ${\hat{v}}_{l_{q}}(t,\text{TE})$ is the learned multi-TE metabolite-dependent basis generated from the training data (described in Section II-B) with order $L_{q}$, and $R_{q}(\cdot )$ represent spatial regularization terms (e.g., weighted-$L_{2}$) for improving the estimation [31] with $\lambda_{q}$ the component dependent regularization parameters. The individual metabolite signal can be synthesized as $\sum_{l_{q}=1}^{L_{q}}c_{l_{q}}^{*}(r){\hat{v}}_{l_{q}}(t,\text{TE})$. It is worth noting that the q-th component may not necessarily be a single molecule but can be a task-specific component. As an example, for metabolite $T_{2}$ mapping, the targeted component in this work was NAA + Cr + Cho and the rest. Thus a component containing only NAA, Cr and Cho can be extracted. The final metabolite maps can be obtained by calculating the voxel-wise $l_{2}$-norm of the spatial coefficients $\left\{c_{l_{q}}^{*}(r)\right\}$. After the separation, the model for $T_{2}$ estimation (Eq. (2)) can be significantly simplified with only one to three molecules considered and thus fewer nonlinear parameters, such as metabolite dependent $T_{2,m}$, $T_{2,m}^{\prime }$ and $\Delta f_{m}$.

For neurotransmitter mapping, a further quantification step can be performed by solving the following problem

(8) min{clglx,clgaba}‖ρ^nt(r,t,TE)−∑lglxclglx(r)vlglx(t,TE)‖−‖∑lgabaclgaba(r)vlgaba(t,TE)‖22+λ1R1(clglx)+λ2R2(clgaba),

where the input ${\hat{\rho }}_{nt}(r,t,\text{TE})$ was obtained by subtracting ${\hat{\rho }}_{met}(r,t,\text{TE})$ from the original reconstruction

(9) $${\hat{\rho }}_{nt}(r,t,\text{TE})=\hat{\rho }(r,t,\text{TE})-{\hat{\rho }}_{met}(r,t,\text{TE}).$$

### E. Accelerated J-Resolved MRSI Acquisition

To achieve high-resolution J-resolved MRSI with the proposed multiparametric processing strategy, we have designed and implemented a new accelerated acquisition.^1^ The proposed acquisition combined a few key features in a multi-spin-echo (SE) MRSI framework (illustrated in Fig. 2). First, we used a combination of a selective excitation pulse and a pair of adiabatic (hyperbolic secant, HS) refocusing pulses for slab selective excitation. As previously described [24], this scheme can achieve reduced chemical-shift displacement errors (CSDEs), better cortical coverage and fewer pulses needed than the sLASER volume selective excitation scheme [23], [37], allowing shorter TEs. We acquired data at the optimized TE values by adjusting the delay between the two refocusing pulses (Fig. 2). Second, we used an EPSI trajectory for rapid spatiospectral encoding with an extended coverage along $k_{x}$ for high resolutions (the echo spacing need not satisfy spectral Nyquist criterion with subspace reconstruction). Third, to extend the coverage in the phase encoding directions ($k_{y}$ and $k_{z}$) without significantly lengthening the scan time, we performed undersampling along $k_{y}$ for each TE. Data for different TEs were acquired sequentially. WET water suppression module [38] was included for weak water suppression with 2 pulses (flip angle = [89, 83]° and an 80 ms delay). Eight outer volume suppression (OVS) bands were applied to suppress the subcutaneous fat (see Supporting Information Fig. S4).

**Fig. 3.**
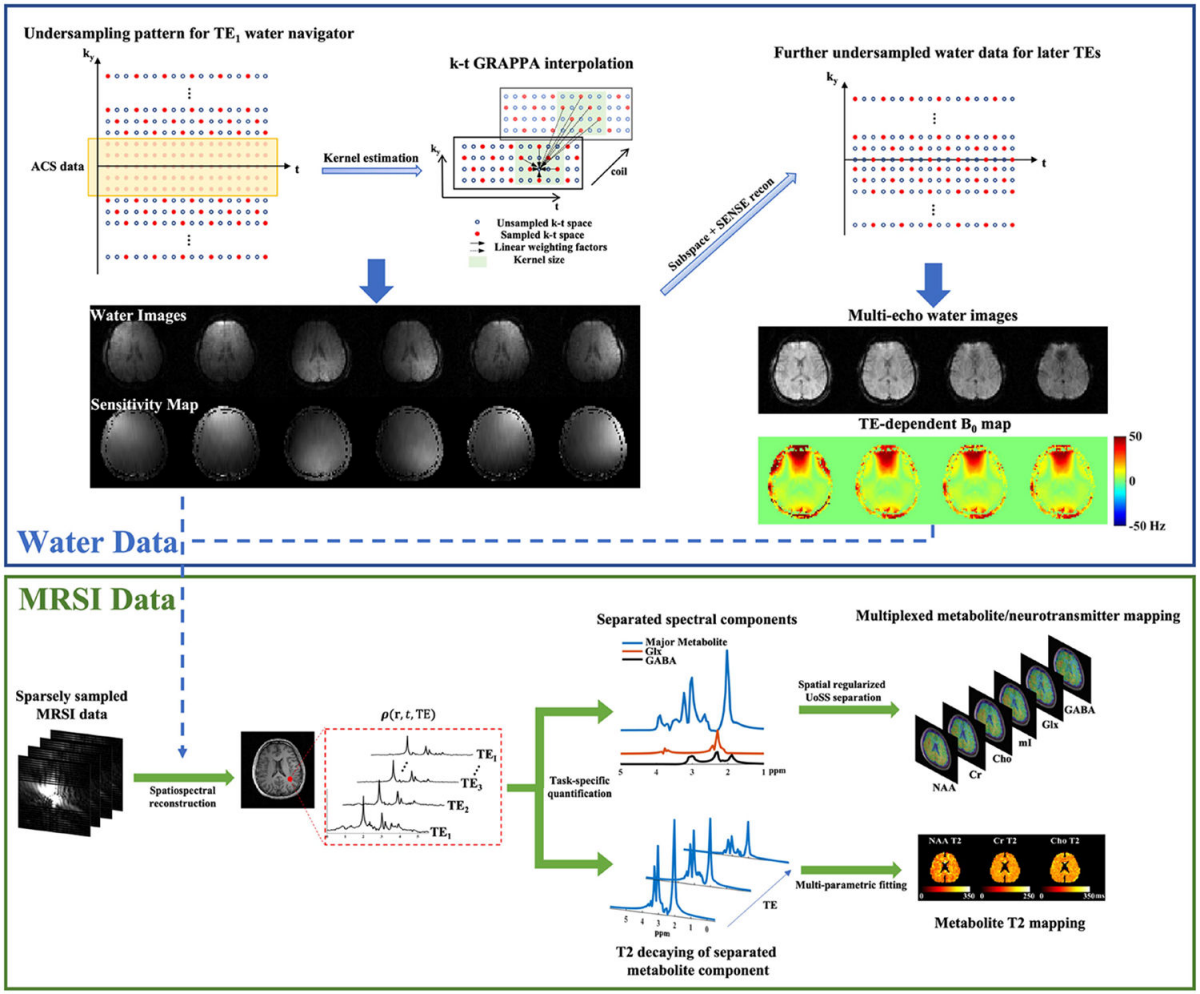
Summary of the proposed data processing and resulting imaging capabilities: (Top panel) Reconstruction of water imaging data; An example of sparsely sampled ($k_{y}$, t)-space and the (k, t)-GRAPPA based interpolation method for the water imaging data acquired at 1st TE were shown in the top panel (left). This step produced fully sampled water images and sensitivity maps for reconstructing later TEs which did not acquire ACS data. Multi-echo water images ($T_{2}^{*}$ weighted) and TE-dependent $B_{0}$ maps can be generated. More details can be found in the texts. (Bottom panel) Multi-parametric molecular imaging from the J-resolved MRSI data; The sensitivity and $B_{0}$ maps from the water data (top panel) were used in the spatiospectral reconstruction, which produced high-quality spatially-resolved multi-TE spectra (column 2). Then the proposed multi-step, task-specific quantification strategy can be performed for simultaneous metabolite/neurotransmitter mapping (top branch) and/or metabolite-specific $T_{2}$ mapping (bottom branch).

Fourth, as the TR can not be significantly shortened for SE-MRSI due to SNR efficiency consideration, we proposed a high-resolution, interleaved water spectroscopic acquisition (Fig. 3 upper panel) to take advantage of the longitudinal recovery time window for auxiliary information (which usually needs separate scans). More specifically, a small-angle water-selective excitation (10°) was applied after the MRSI encoding module (minimal perturbation to the metabolite signals), which was first followed by a readout at the k-space center and then a high-resolution encoding module. The k-space center navigator was used to extract field drifts and monitor motion-induced phase variations during acquisition. The high-resolution encoding contains an EPSI readout with blip gradients along $k_{y}$ (can include $k_{z}$ as well) [33], [39] and a bigger k-space coverage than the preceding MRSI encoding. This resulted in an undersampled ($k_{y}$, t)-space, i.e., 3× undersampling with 2 $k_{y}$ blips (Figs. 2 and 3 upper panel). To facilitate the reconstruction, we also designed a multi-TE sampling strategy. This included a fully-sampled auto-calibration (AC) data at the first TE (i.e., 15 center $k_{y}’s$ for a 64×64×10 matrix size) and undersampled data only for the remaining TEs. As a result, the resolution of the water data typically has a 2 to 2.5× larger matrix size than the J-resolved MRSI data. Finally, note that in order to keep the total number of excitations the same across TEs, the acquisition of AC data at the first TE will have a slightly smaller k-space coverage than other TEs.

### F. Other Processing Details

Fig. 3 summarizes the major data processing steps and the resulting imaging capabilities. Additional explanations on specific steps are provided here.

#### 1) Reconstruction of Water Images:

We interpolated the undersampled (k, t)-space corresponding to the interleaved water imaging data and generated high-quality auxiliary information for MRSI data processing. Specifically, we first performed a (k, t)-GRAPPA reconstruction to interpolate the data for the first TE using a 7 × 9 ($k_{y}$, t) kernel, from which a set of coil sensitivity maps and water signal subspace can be derived. Assuming invariance of coil sensitivity and water subspace across TEs, a joint SENSE and subspace reconstruction can be applied to the water data for the remaining TEs. This strategy faithfully reconstructed the water spectroscopic signals, from which auxiliary information such as TE-dependent $B_{0}$ maps and anatomical images at different gradient echo times can be obtained (see Fig. 3, upper panel) and used for subsequent spatiospectral processing. The ESPIRiT method was used to estimate coil sensitivity maps [40].

#### 2) Nuisance water/lipid Removal and Spatiospectral Reconstruction:

SENSE reconstruction using sensitivity maps estimated from the water images was performed to combine the undersampled multi-coil J-resolved MRSI data. Subsequently, nuisance water/lipid signal was removed from the coil-combined data using a union-of-subspace-based method [24], [32]. This strategy offers a lower computation burden than removing nuisance signals coil by coil.

After nuisance removal, the desired spatio-temporal-TE function, ρ(r,t,TE), can be reconstructed using a previously investigated augmented subspace constrained reconstruction method, incorporating the learned subspace/basis and a spatial constraint (i.e., a weighted finite difference regularization with weights derived from the accompanying water images). As the temporal basis has full spectral bandwidth (BW) and high spectral resolution, and may not be defined on the same temporal grids as the MRSI data, a temporal interpolation of the basis can be done to grids at an integer multiple of the half EPSI echospacing for subspace fitting. These processes and the subspace reconstruction algorithm have been discussed at length previously, e.g., Refs. [24], [32], [33], thus not presented in details here. To further account for the discrepancy between experiment-dependent lineshape distortion and the spectral variations captured by the learned subspace, a subspace adaptation scheme was used which is described in the section below.

#### 3) Subspace Adaptation:

While the learned subspace offers significant dimensionality reduction while capturing realistic variations of metabolite signals across TE, it may not completely capture subject and experiment-dependent lineshape variations. Our subspace adaption scheme addressed this issue. Specifically, we used a lower-resolution version of the J-resolved MRSI data for this step (e.g., 24 × 24 × 8 matrix size with an initial $B_{0}$ correction). These data can be from either k-space truncation of the high-resolution data or previously acquired training data. These higher-SNR data were first projected onto the learned subspace, generating a set of voxel-wise reference signals $\rho_{ref}(r,t,\text{TE})$. These references were then refitted to the low-resolution experimental data $\rho_{lr}(r,t,\text{TE})$ with a lineshape adaptation term modeled by a low-order FIR filter, i.e.,

(10) $$\rho_{lr}(r,t,\text{TE})=\rho_{ref}(r,t,\text{TE})\times \sum_{p=1}^{P}a_{p}(r,\text{TE})\;\text{exp}(i2\pi p\delta ft),$$

where $\rho_{ref}(\cdot )$ is the voxel-dependent reference from the subspace projection. This model is equivalent to a spectral domain convolution and accounts for residual intravoxel field inhomogeneity-induced lineshape distortion by considering multiple frequency components at a grid of δf (chosen as the spectral resolution of the basis). P were chosen from 12 to 16. The refitted data were then formed into a Casorati matrix to extract a new set of adapted $\left\{v_{l_{m}}^{\left(a\right)}(t,\text{TE})\right\}$ for spatiospectral reconstruction of ρ(r,t,TE). This adapted subspace offers a more accurate representation of in vivo data, as shown in Supporting Information Fig. S3.

To account for the macromolecule (MM) signals in the shortest TE acquired for metabolite $T_{2}$ mapping, we incorporated a learned MM basis as described in Refs. [26], [28] into the reconstruction and removed the fitted MM component from the data before the proposed quantification steps.

## III. Experimental Setup

### A. Simulation

A multi-TE computational MRSI phantom (64 × 64 voxels) was constructed to validate the proposed TE optimization and quantification strategies. Specifically, spectra of different molecules were generated using Eq. (2) with tissue-specific literature parameter values and combined with spatially varying tissue fractions from segmentation of anatomical images. White Gaussian noise at a practical SNR level (i.e., SNR = 20) defined w.r.t. the NAA peak height was added for Monte-Carlo simulations (N = 100) to assess estimation bias and variance. For metabolite/neurotransmitter separation, we compared our optimal 2 TEs (65 and 80 ms) against single short TE (35 ms) with 2 averages, and shortest 2 TEs (35 and 50 ms) under an equivalent scan time constraint. For metabolite $T_{2}$ estimation, a literature 4-TE choice (50, 100, 160 and 220 ms) and our optimal 4 TEs (35, 200, 245, 275 ms) were compared.

### B. Phantom Studies

Both phantom and in vivo experiments were performed to evaluate the proposed method. All experiments were conducted on a Siemens Prisma 3 T scanner (Siemens Healthineers, Erlangen, Germany). A customized metabolite phantom was built with a set of brain metabolites dissolved in individual conical tubes. For phantom imaging, six tubes containing 100 mM NAA, 100 mM Cr, 100 mM Cho, 100 mM Glu, 200 mM GABA and 200 mM mI were made and imaged. TE-dependent molecule-specific basis was extracted from high-SNR data acquired for each high-concentration tube, and used for TE optimization and subsequent reconstruction.

Another set of three tubes were made with mixtures of metabolites at physiological concentrations (Fig. 5), i.e., Tube 1 (Mix 1): 12 mM NAA, 8 mM Cr, 3 mM Cho, 6 mM mI, 4 mM Glu and 3 mM GABA; Tube 2 (Mix 2): 12 mM NAA, 8 mM Cr, 3 mM Cho, 6 mM mI, 8 mM Glu and 2 mM GABA; Tube 3 (Mix 3): 12 mM NAA, 8 mM Cr, 3 mM Cho, 6 mM mI, 12 mM Glu and 1 mM GABA. Multi-TE MRSI data from this three-tube phantom were acquired to validate our acquisition and metabolite/neurotransmitter separation methods, with FOV = 140 × 140 × 10 mm^3^, matrix size = 40 × 40 (in-plane resolution of 3.5 mm × 3.5 mm), spectral bandwidth = 925.92 Hz with 300 FID samples, and optimized TE returned by our analysis as well as standard short TE (i.e., 40 ms).

### C. In Vivo Studies

Approval from the local Institutional Review Board (IRB) and participant consents were obtained for all in vivo experiments (protocol number: 21074, approval date: July 15th, 2022). We collected brain J-resolved MRSI data from healthy volunteers to demonstrate our simultaneous metabolite/neurotransmitter and metabolite $T_{2}$ mapping capabilities. The two TEs for optimized metabolite and neurotransmitter separation were 65 and 80 ms, and four TEs optimized for metabolite $T_{2}$ mapping were 35, 200, 245, and 275 ms. The other key parameters were: 220 × 220 × 64 mm^3^ FOV, 64 × 64 × 10 matrix size (3.4 × 3.4 × 6.4 mm^3^ voxels), 1.18 ms echospacing, 300 echoes and 2 × $k_{y}$ undersampling with the center 20 × 10 ($k_{y}$ × $k_{z}$) fully sampled and an elliptical sampling pattern (an overall 52.97% sampling).

The total scan time for a 2-TE dataset was ~14.4 mins with TR = 1.2 s, and ~25.8 mins for a 4-TE dataset with TR = 1.1 s. For each in vivo scan, we also acquired anatomical images using MPRAGE with 240 × 240×192 mm^3^ FOV (sagittal), 256 × 256 × 192 matrix size (isotropic 1 mm resolution) and TR/TE = 1.8 s/3.27 ms (~4.5 mins). Lower resolution data (matrix size of 32 × 32 × 8 and echo spacing of 0.8 ms) using the same TE combinations were collected multiple times at each TE combination to provide data for subspace adaptation. Test-retest high-resolution scans were conducted to evaluate the repeatability of metabolite/neurotransmitter and $T_{2}$ estimation, respectively.

A PTE patient dataset was also collected to assess the potential of our method for clinical applications (local IRB protocol number: 20520, approval date: March 24th, 2022), with the following acquisition parameters: TR/TE = 1000/[65,80] ms, FOV = 220 × 220 × 64 mm^3^, and matrix size = 42 × 42 × 8 (5.2 × 5.2 × 8 mm^3^ voxel size), 0.92 ms echospacing, and 200 echoes. The resolution for the patient data was lower than healthy volunteers for patient comfort and minimizing motion. The MRSI acquisition was ×9 mins. FLAIR images (isotropic 1 mm resolution) were also acquired, which can be used to identify abnormal tissues (i.e., regions with hyperintensity).

## IV. Results

### A. Simulation Results

**Fig. 4.**
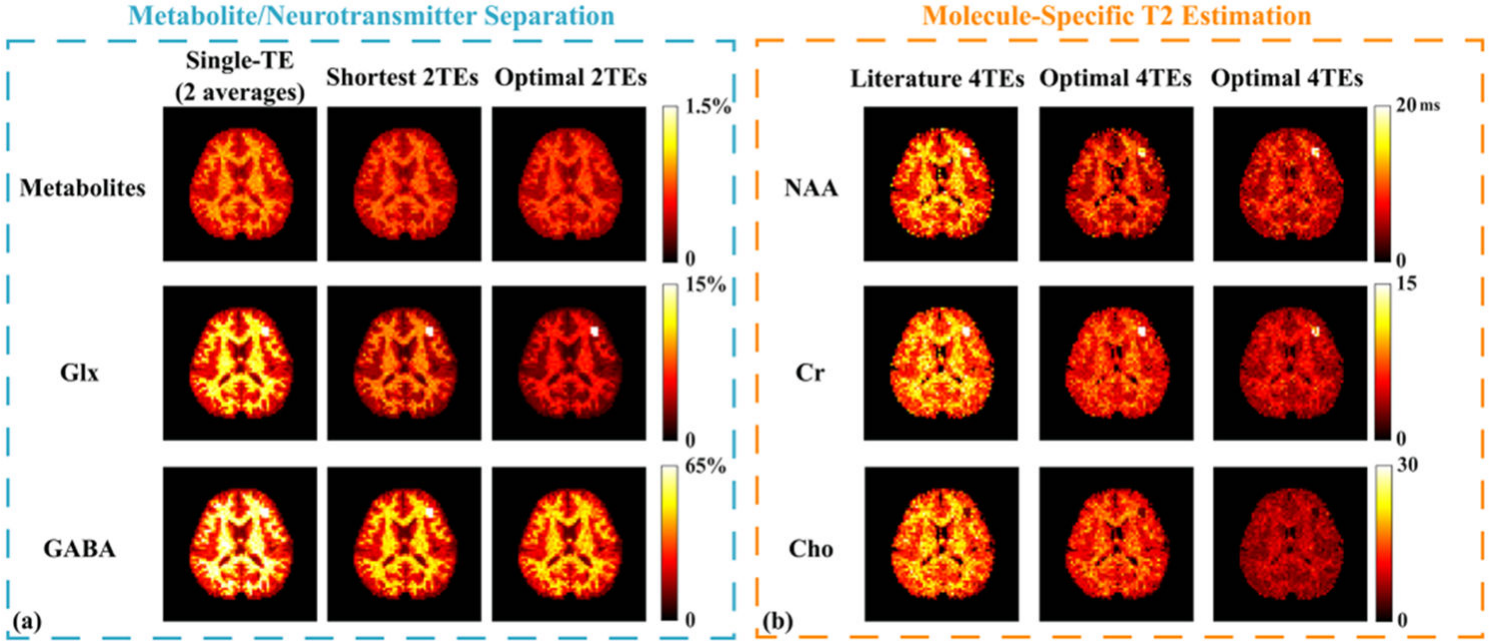
Monte-Carlo simulation results validating the optimal experiment designs: (a) Standard deviation (SD) maps for metabolite and neurotransmitter estimates from different Te choices, The SD maps were normalized w.r.t. the concentrations of each component. Cases considered were all 2 TEs, i.e., single short TE with 2 averages (35 ms; column 1), shortest 2 TEs (35 and 50 ms; column 2) and proposed optimal 2 TEs (65 and 80 ms; column 3), with quantification done with the proposed method. Smallest SDs were achieved using the optimal 2 TEs, with more significant improvement for GABA and Glx. (b) SD maps for metabolite $T_{2}$ estimates (in ms), i.e., NAA (row 1), Cr (row 2) and Cho (row 3) from different scenarios. The first two columns show results from different 4-TE combinations but the same parametric fitting. Significantly reduced variance was achieved by TE optimization (column 2). Our proposed multi-step quantification strategy further reduced the estimation variances with the same optimized TEs (column 3).

Standard deviation (SD) maps from the Monte-Carlo simulations were shown in percentage (normalized to the true values) in Fig. 4(a) for molecular separation and 4(b) for $T_{2}$ estimation, respectively. Lowest SDs were achieved using the optimal 2 TEs (column 3 in Fig. 4(a)) for all components, with more significant improvement for GABA and Glx, which validates our proposed TE optimization. For metabolite $T_{2}$ estimation, the optimal 4 TEs (column 2 in Fig. 4(b)) produced apparently lower SDs compared to the literature 4 TEs (column 1) when using the same parametric model fitting, consistent with our estimation-theoretic prediction. The estimation variances were further reduced by our proposed multi-step quantification with the same optimized 4 TEs (column 3 in Fig. 4(b)). While the optimal TE choice is SNR independent, the absolute estimation variance scales linearly w.r.t. noise variance. The acceptable variance can be user and application dependent, and can be estimated by combining our analysis with estimated noise level and test-retest experiments.

### B. Phantom Results

For phantom imaging, we obtained an optimized TE of 80 ms (minimum CRLB) for Glu and GABA estimation using the experimentally obtained molecule-specific basis. As Glu and GABA were the components of interest here (more challenging ones), NAA, Cr, Cho and mI were combined into a single component during the quantification process. The optimal TE for both Glu and GABA is similar to the choice for in vivo case. For both phantom and in vivo data, we first derived frequency and phase drifts from the center k-space navigators and used them to correct both the Multi-TE MRSI and water imaging data (more details can be found in [24]).

**Fig. 5.**
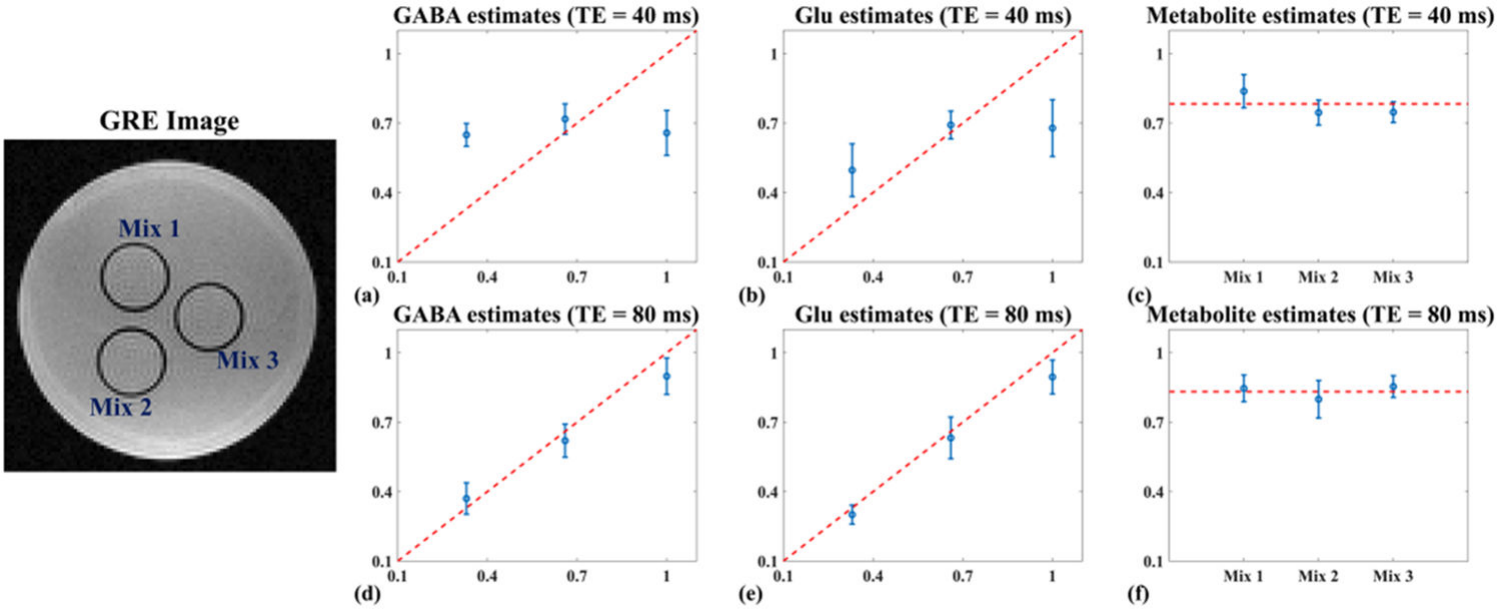
Phantom results for Glu and GABA estimation with TE optimization: The phantom setup was shown in the GRE image on the left (with three mixtures labeled). (a)–(c) and (d)–(f) show regressions of estimated Glu, GABA and metabolites (NAA, Cr, Cho and ml combined) against the ground truth, for 40 ms TE (a-c; a commonly used short TE) and 80 ms TE (d-f; optimized TE), respectively. The Glu, GABA and metabolite estimates were normalized to the highest concentration among the three mixtures (so was the ground truth). In (a), (b), (d), and (e), an identity line was plotted (red) as the ideal result. As the metabolite concentrations were the same in all mixtures, mean value was plotted as the red horizontal lines in (c) and (f). Estimation standard deviations were shown as the error bars in the plots. The estimated molecular concentration ratios matched well with the ground truth for the proposed method with the optimized 80 ms TE, significantly better than the short-TE results.

Fig. 5 shows a set of quantification results from the phantom data. The GRE image on the left illustrates the tube arrangement with different mixtures. Both receive $B_{1}$- and transmit $B_{1}$ factor were corrected using water signals as a reference from a nonwater suppressed scan with the same acquisition parameters. The optimized TE of 80 ms produced more accurate estimates of Glu and GABA across different tubes with varying concentrations than simply choosing a short TE of 40 ms, validating the proposed TE optimization and quantification strategies. Apparent variance reduction can be observed (Fig. 5(a), (b), (d), (e)), i.e., variances of Glu and GABA reduced by 52% and 19%, respectively (~ 40% overall). Note that since we are using an institutional unit, we normalized the estimated concentrations to the highest value for both the estimates and ground truth. For major metabolites, the mean values within three mixtures were plotted as red dashes in (c) and (f) as a “ground truth” reference. The major metabolite estimation maintained a similar performance for different TE choices. A larger bias can also be observed for GABA and Glu estimates from the 40 ms TE data.

### C. In Vivo Results

**Fig. 6.**
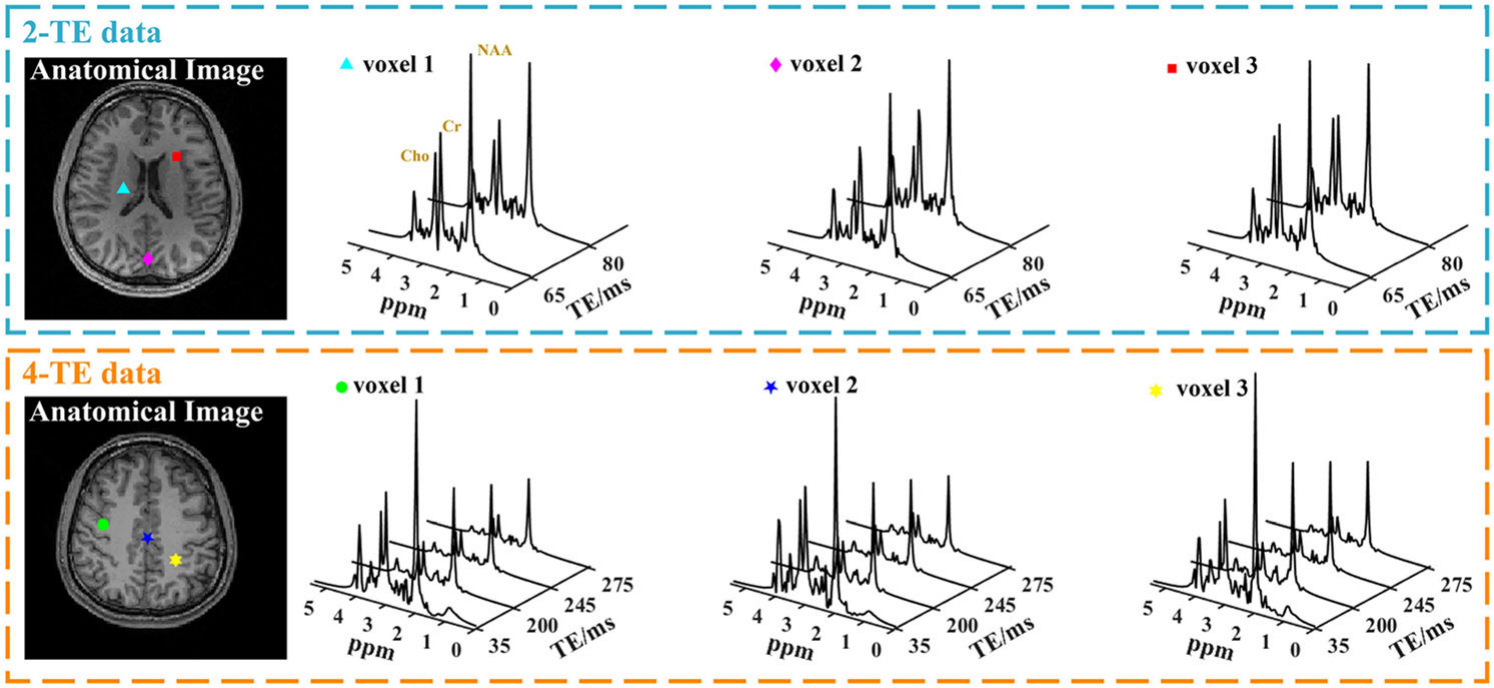
In vivo spatiospectral reconstruction results for different TE combinations (2-TE data shown on top and 4-TE data below). Anatomical images ($T_{1}$ weighted) are shown in the first column, and multi-TE spectra from different voxels are shown in the subsequent columns (3.4 × 3.4 × 6.4 mm^3^). Voxel locations were identified by markers of different shapes and colors in the anatomical images. High-quality spectra were obtained using the proposed learned and adapted subspace. Clear TE-dependent spectral features, e.g., J-coupling-induced differences and $T_{2}$ decays can be visualized.

**Fig. 7.**
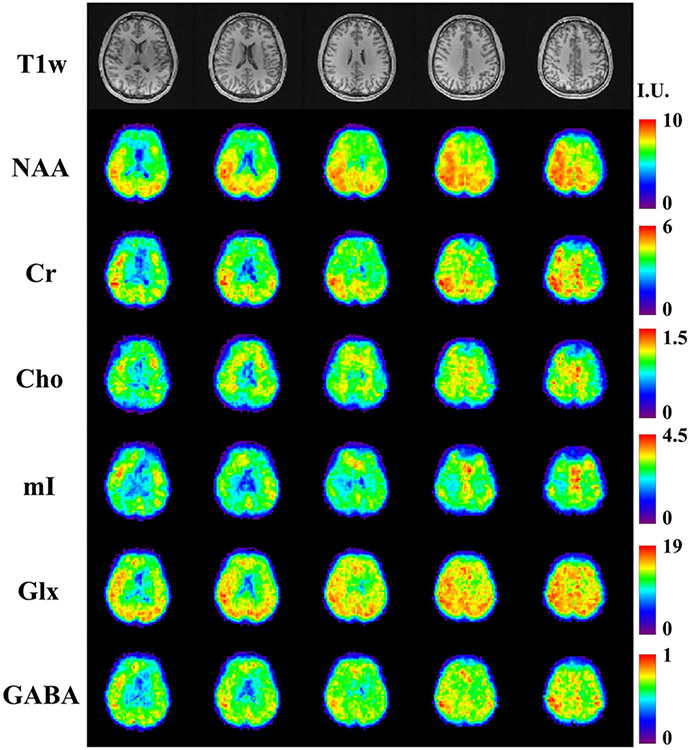
In vivo high-resolution, 3D metabolite and neurotransmitter mapping results (healthy volunteer): Anatomical images (T1w) for different slices across the imaging volume are shown in the first row. The maps of NAA, Cr, Cho, ml, Glx and GABA for the corresponding slices are shown in the subsequent rows with an institutional unit (I.U.). Different contrasts across tissue types can be visualized for different metabolites.

**Fig. 8.**
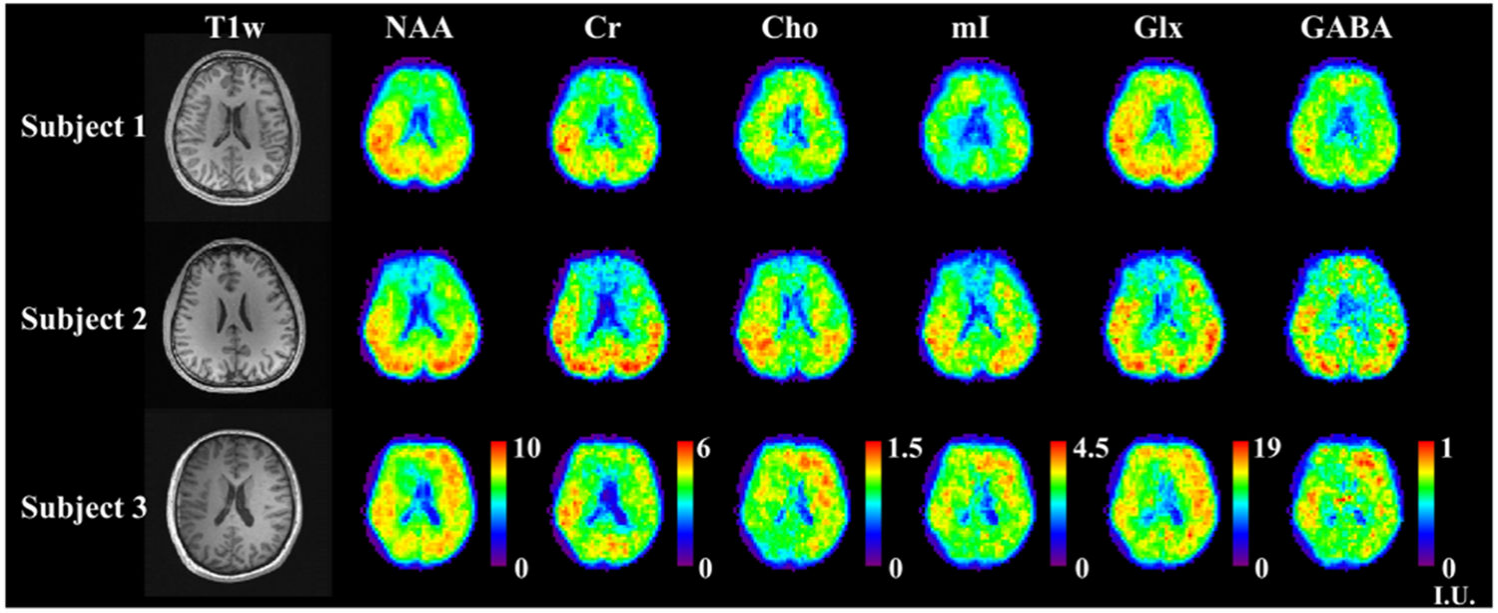
High-resolution metabolite and neurotransmitter maps from multiple volunteers (displayed in different rows). Anatomical image are shown on the left of each row, with metabolite maps in subsequent columns in the same institutional unit as in Fig. 7 across subjects.

**Fig. 9.**
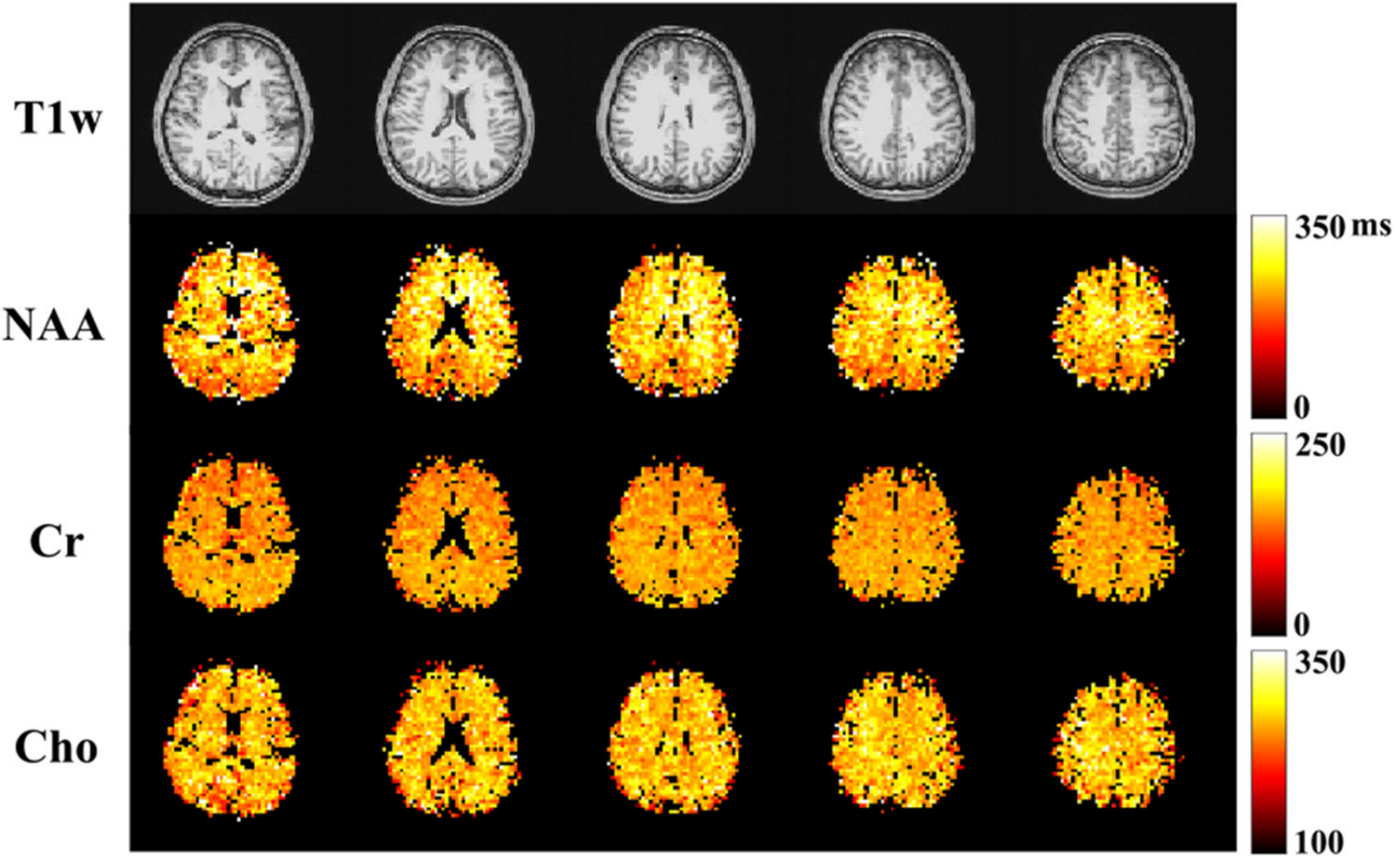
High-resolution 3D metabolite $T_{2}$ mapping using an optimal 4-TE acquisition from a healthy volunteer. Again, $T_{1}$ weighted water images (T1w) from different slices are shown in row 1, and $T_{2}$ maps of NAA, Cr, and Cho produced by the proposed method in rows 2–4. CSF-dominant voxels were excluded for $T_{2}$ fitting. White/gray matter contrast can be visualized in the NAA $T_{2}$ maps while less contrast for Cr $T_{2}$ maps, consistent with previous reports [12], [41].

**Fig. 10.**
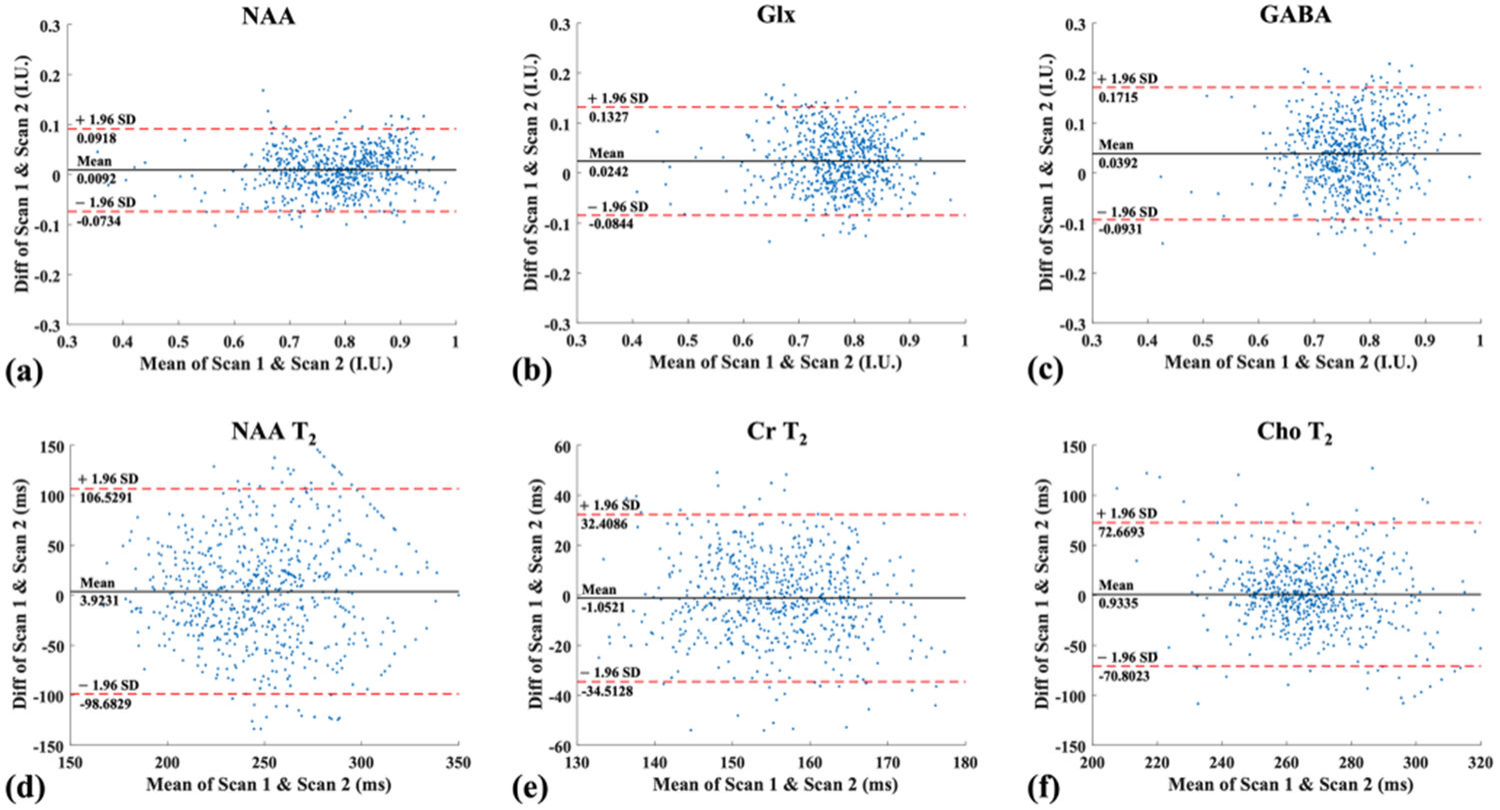
Bland-Altman analysis using test-retest scans for metabolite/neurotransmitter mapping (a)–(c) and metabolite $T_{2}$ mapping (d)–(f). In each plot, the measurement differences (scan 1 vs. scan 2) for all voxels were plotted against their means. The horizontal red dash lines indicate ± 1.96 × SD and the mean difference was displayed as the black solid line. (a)–(c) show the results for NAA (a), Glx (b) and GABA (c). Note that the estimated “concentrations” for each molecule were normalized individually for this analysis; (d)–(f) show the analysis for $T_{2}’s$ estimates of NAA (d), Cr (e) and Cho (f). As can be seen, a high level of reproducibility was achieved for both tasks.

**Fig. 11.**
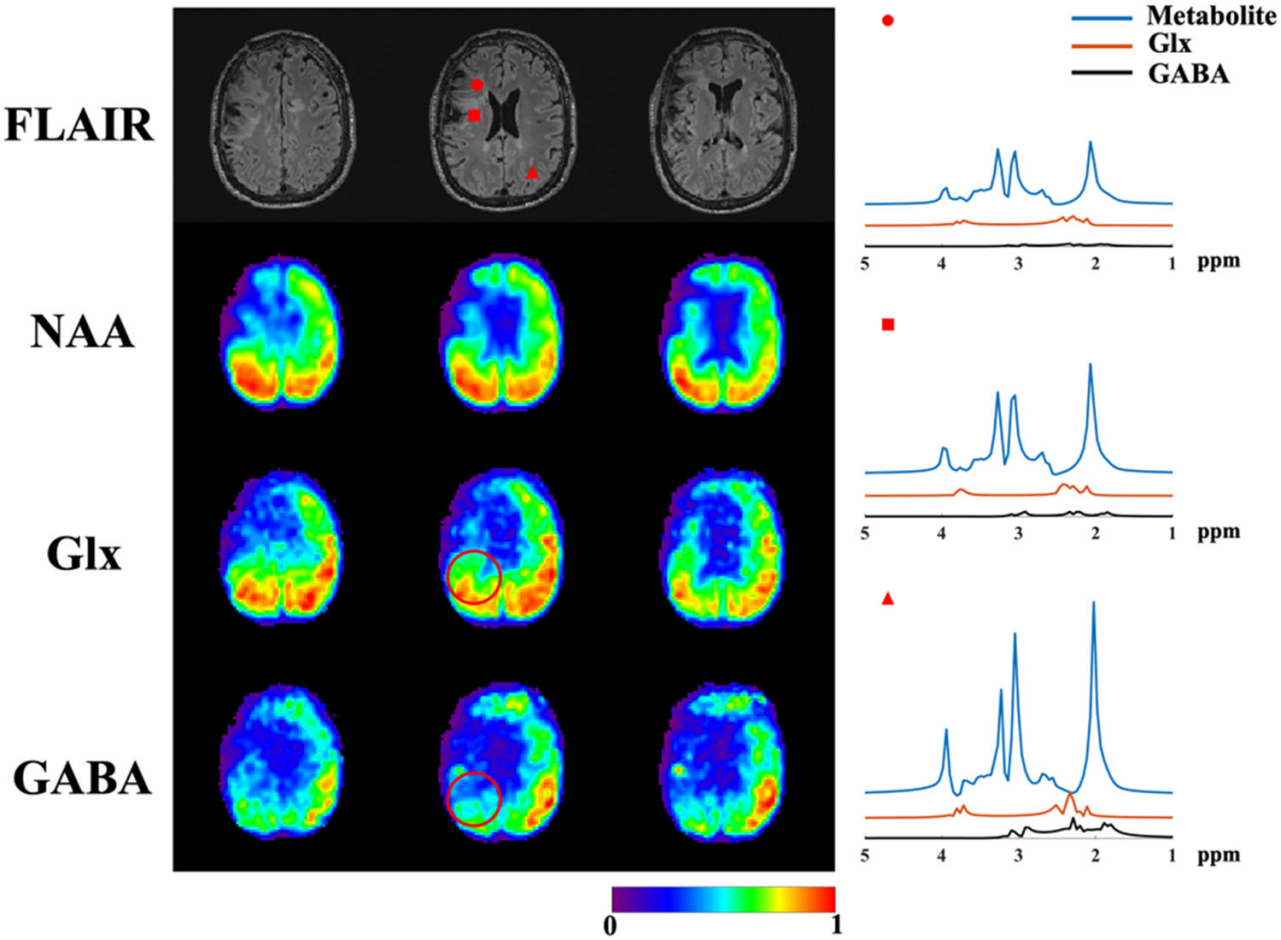
Simultaneous metabolite and neurotransmitter mapping for a PTE patient. FLAIR images are shown in the first row of the left panel, followed by molecular maps for the corresponding slices. Spatially-resolved metabolite and neurotransmitter spectral components (in different colors) from three different voxels are shown in the right panel, two from “hyper-intensity” lesions (red dot and red square) and one from normal-appearing tissue (red triangle). Each map (left panel) was normalized individually across the imaging volume to better visualize the molecule-specific spatial patterns. Molecule-specific abnormality can be observed in the multi-molecular maps, e.g., differences in the Glx and GABA distributions in the region indicated by the red circle.

Representative in vivo results from both healthy volunteers and PTE patients are shown in Figs. 6, 7, 8, 9, 10, and 11. As shown in Fig. 6, high-quality spatially-resolved TE-dependent spectra were produced by the proposed method from both the 2-TE and 4-TE acquisitions, with nominal 0.06 cc voxels. TE-dependent spectral variations from different metabolites, such as $T_{2}$ decays and J-coupling induced changes, can be clearly observed in the spatiospectral reconstruction. As the learned and experimental lineshape adapted subspace was explicitly enforced during the reconstruction (fidelity of subspace demonstrated in Supporting Information Fig. S3), the reconstructed multi-TE spectra “appear” noiseless. But the spatial uncertainty due to measurement noise will be absorbed into the spatial coefficients.^2^

A set of 3D high-resolution metabolite and neurotransmitter mapping results from a healthy volunteer are shown in Fig. 7. The optimized 2-TE combination (65 and 80 ms) targeting metabolite and neurotransmitter separation was used for data acquisition. $T_{1}$-weighted images (MPRAGE) of multiple slices across the imaging volume were displayed in the first row. Separated metabolite (NAA, Cr, Cho and mI) and neurotransmitter (Glx and GABA) components for the corresponding slices were quantified using the proposed multi-step strategy, and shown in the subsequent rows, respectively. The maps were normalized across the slices for each molecule individually, and revealed interesting and biologically meaningful molecule-dependent spatial distributions. For example, the ventricles exhibited low intensities consistently across different molecules, as expected due to the low abundance of metabolites. The gray-matter-rich regions in the upper slices showed higher levels for most metabolites. Stronger signals in gray matter (GM) than white matter (WM) for Cr, Glx and GABA, and higher concentrations for Cho in WM dominant tissues can be visualized from the quantified maps, which are consistent with results from previous studies [24]. Fig. 8 shows high-resolution metabolite and neurotransmitter mapping results (at a similar slice location) from multiple healthy volunteers, demonstrating good inter-subject robustness. Results for more slices can be found in Supporting Information Figs. S5 and S6.

Fig. 9 shows a set of high-resolution metabolite $T_{2}$ maps from the proposed method (3.4 × 3.4 × 6.4 mm^3^ nominal voxels). The results were produced by a 4-TE acquisition (35, 200, 245 and 275 ms) optimized for $T_{2}$ estimation of NAA, Cr and Cho. Voxels with CSF fraction greater than 50% were not selected for $T_{2}$ fitting due to low and unreliable metabolite estimates in these regions. As can be seen, greater $T_{2}$ values of NAA can be observed in the WM across different slices. For Cr and Cho, no significant differences in $T_{2}’s$ were observed between GM and WM, which is consistent with literature finding [41]. The averaged metabolite $T_{2}’s$ in different brain tissue types (for the same subject) are shown in Supporting Information Table S1. The measured NAA $T_{2}$ is 258.51 ± 46.33 ms in WM and 226.55 ± 49.90 ms in GM, Cr $T_{2}$ is 155.35 ± 13.83 ms in WM and 151.09 ± 15.93 ms in GM, consistent with literature values [12], [41], [42]. Cho $T_{2}$ is 265.29 ± 21.39 ms in WM and 259.46 ± 36.90 ms in GM, similar to values reported in [42]. $T_{2}$ mapping results from more subjects can be found in Supporting Information Figs. S7 and S8. Regional values summarized across different subjects can be found in Supporting Information Table S2. The comparison of metabolite $T_{2}$ estimates between optimal 4-TE and literature 4-TE acquisitions shows ~60% variance reduction for NAA, Cr and Cho $T_{2}’s$ (Supporting Information Fig. S9).

We have also evaluated the reproducibility of our method using test-retest scans and a Bland-Altman analysis (Fig. 10) of the two repeated 2-TE scans for metabolite and neurotransmitter mapping (first row), and two repeated 4-TE scans for metabolite $T_{2}$ mapping (second row). Limit of agreements are shown as a quantitative measure of the consistency between repeated measurements. A high level of consistency between the repeated scans can be obtained using the proposed method for differen acquisitions and estimation tasks.

Fig. 11 shows a set of results from a PTE patient. High-resolution metabolite (NAA as an example here) and neurotransmitter (Glx and GABA) maps can be simultaneously obtained using the proposed method. Molecule-dependen tissue abnormality can be observed in the multi-molecular maps, e.g., lower concentration in the “dead” and surrounding “hyper-intensity” (injured) tissues, while higher concentration in normal-appearing tissues. Separated component-specific spectra from representative voxels (labeled by different markers in the FLAIR image) were displayed on the right panel. Spectra differences from different components can be visualized, with noticeable heterogeneity even within the same anatomical “hyper-intensity” region (indicated by the red dot and square). Furthermore, unique alteration of excitatory (Glx) and inhibitory neurotransmitters (GABA) was revealed by the proposed method (e.g., see the region marked by the red circles in Fig. 11). There seems to be a larger region of GABA reduction and stronger asymmetry in the spatial distribution compared to other molecules, which is particularly interesting considering that this patient suffers from seizures after the injury. Such information can not be obtained from anatomical images. Evaluations on more patients are currently ongoing and will be reported in a subsequent publication.

## V. Discussion

We demonstrated the feasibility of high-resolution, 3D multi-parametric molecular imaging of the brain by integrating SNR-efficient rapid spatiospectral encoding, task-specific optimal experiment design, subspace-based reconstruction and quantification. Compared to the previous work (Ref, [24]), a novel task-specific optimal experimental design and a spectral quantification method under the subspace framework were proposed, which improved performance in both metabolite/neurotransmitter separation and molecular $T_{2}$ estimations. We also extended the acquisition by incorporating a more aggressive (k,t)-space undersampling design and advanced reconstruction for water navigators, which provide useful information (i.e., anatomical information, field inhomogeneity, coil sensitivity) at a higher spatial resolution. In this work, we validated the new method quantitatively via simulation and phantom experiments. High-SNR metabolite/neurotransmitter maps were obtained from both healthy volunteers and PTE patients, which were not shown before. High-resolution metabolite $T_{2}$ maps with the best combination of spatial resolution and volume coverage were obtained with good reproducibility.

Our estimation-theoretic analysis under the augmented subspace imaging framework provides a general avenue for task-specific experiment optimization, e.g., targeting specific parameters (e.g., concentration and/or relaxation parameters) of a molecule and/or a specific subset of molecules of interest. This strategy can be tailored to different applications. The multi-TE metabolite subspaces generated for the optimization can be flexibly adapted to different sequences with appropriate modifications to the QM simulations. While we demonstrated in this work optimized TE selections for separate tasks, i.e., metabolite and neurotransmitter signal separation and metabolite $T_{2}$ estimation, we note that alternative joint optimization for different sets of parameters, or reconstructing full 2D J-resolved spectra, can be done. For example, depending on the importance of different measures for different applications, one can first choose optimized TE for estimating the concentration of specific molecules, and then include additional TEs optimized for $T_{2}$ estimation. This may slightly compromise the performance compared to optimized TEs for a single task. When optimizing for a group of molecules, the CRLB was calculated by summing corresponding diagonal elements in the iFIM, which may be biased towards components with significantly larger variances than others. A weighting strategy may be considered in future work.

Macromolecules are an important component to consider for data acquired at shorter TEs. To consider this factor, we compared TE optimizations for Glx and GABA with and without the presence of MM signals (Fig. S1). The CRLBs for both Glx and GABA component increased slightly when considering MMs in the model, as expected. However, the optimal TE combinations for both Glx and GABA did not change when adding an MM component into the augmented subspace model. This was expected as our original optimal design already favored medium TEs and macromolecules have short $T_{2}’s$. Note that our proposed TE optimization characterizes an “averaged” performance across a large range of spectral parameter values (e.g., for spectral linewidth, frequency shift, and concentration etc), thus informative for most practical experimental conditions. However, the current TE optimization can be limited by mismatch between the experiment-dependent lineshape distortion and the parametric model used for training FIDs generation. For higher-resolution acquisitions with lower SNR, the estimation variance can still be too high even with TE optimization. In this case, reconstruction at a lower resolution for the weaker components may be considered.

The current multi-step estimation strategy provides a robust way to extract a number of parameters of interest, which can be generalized to more metabolite components. The proposed multi-step estimation strategy helped mitigate the over/underestimation issue for the weaker signal components (i.e., Glx and GABA in this work) compared to one-step direct UoSS-based separation. Meanwhile, there is room for further improvements. The initial VARPRO-based parametric fitting is inherently sensitive to model mismatch and can converge to an undesirable local minimum. To this end, more complicated parametric models and optimization strategies can be considered. Recently, learning-based methods [43], [44] have been described and shown promise for fast and robust single-TE quantification. Methods that incorporate multi-TE data with potential joint estimation and experiment design optimization are worth investigating in future research. Note that the estimation of metabolite $T_{2}$ may also be affected by the performance of molecular component separation and the handling of macromolecule signals at short TEs (i.e., TE = 35 ms), for which further optimization may be explored. The accuracy of tissue-specific molecular $T_{2}$ values will be more carefully validated in future research, e.g., comparisons against single-voxel spectroscopy measurements.

It is possible to further accelerate the MRSI data acquisition using more aggressive undersampling with complementary sparse sampling across TEs. The parallel imaging reconstruction for MRSI data was done TE by TE, but can be extended to jointly interpolate multi-TE data. More advanced spatial/spectral constraints such as learned nonlinear low-dimensional manifolds [28], [45] and generative-image-model-based spatial constraints [46] will be developed to enhance the reconstruction performance. The current water imaging data acquisition can also be further enhanced with higher-resolution encoding (e.g., blips along both $k_{y}$ and $k_{z}$) and additional preparation modules ($T_{1}$/$T_{2}$ preparation or diffusion encoding modules [47]) to generate more imaging contrasts as well as richer quantitative imaging capability. Moreover, multi-slab or simultaneous multi-slice excitation strategies [48], [49], [50] will be incorporated in subsequent publications to demonstrate further accelerated acquisition and larger brain coverage. More sophisticated gradient designs (e.g., flow-compensated gradients [51]) and additional motion correction schemes [52] may be used to generate data with higher quality.

## VI. Conclusion

We presented a novel approach for multi-parametric molecular imaging of the brain via optimized, accelerated J-resolved ^1^H-MRSI. Simulation and phantom results validated the effectiveness of the proposed method. High-resolution, simultaneous metabolite/neurotransmitter mapping and metabolite $T_{2}$ mapping can be achieved within clinically feasible times. The proposed method has the potential to provide richer information for revealing and understanding metabolic alterations in neurological diseases.

## Supplementary Material

supp1-3349375

## Footnotes

1 The proposed experiment optimization and processing strategies can be readily adapted for different acquisitions, such as low-resolution CSI or EPSI scans.

2 Recall that the training data for subspace learning were generated using a parametric biophysical model thus noiseless.
