# Supplementary material for: Multi-Parametric Molecular Imaging of the Brain Using Optimized Multi-TE Subspace MRSI
Source: IEEE Trans Biomed Eng. Author manuscript; Available in PMC 2024 Jun 7. (PMC11160977; doi:10.1109/TBME.2023.3349375)
Supplement: supp1-3349375 [file NIHMS1995940-supplement-supp1-3349375.pdf]

Supplementary Materials

Fisher Information Matrix (FIM) Calculation for the Multi-TE Parametric Model

We show here that how the FIM for the multi-TE parametric spectroscopic signal model can be derived. Specifically, The noisy multi-TE FID signal can be described as:

$$s(n,\mathrm{TE}) = \hat{s}(n,\mathrm{TE};\boldsymbol{\theta}) + \xi(n,\mathrm{TE}), \tag{1}$$

where $s(n,\mathrm{TE})$, $\hat{s}(n,\mathrm{TE};\boldsymbol{\theta})$ and $\xi(n,\mathrm{TE})$ denote the measured FID signals, model prediction by Eq. (2) in the main text and white Gaussian noise with a standard deviation δ, respectively. The variables n and TE index FID sampling point and TE. We defined the set of unknown spectral parameters $\boldsymbol{\theta}$ as:

$$\boldsymbol{\theta} = [\mathbf{c};\mathbf{T_2};\mathbf{T_2'};\boldsymbol{\Delta}\mathbf{f};\varphi;\mathbf{g}], \tag{2}$$

with each vectorized component being

$$\begin{aligned}
\mathbf{c} &= [c_1,\ldots c_M]^T, \\
\mathbf{T_2} &= [T_{2,1},\ldots,T_{2,M}]^T, \\
\mathbf{T_2'} &= \left[T'_{2,1},\ldots,T'_{2,M}\right]^T, \\
\boldsymbol{\Delta}\mathbf{f} &= [\Delta f_1,\ldots,\Delta f_M]^T, \\
\varphi &= [\varphi_{\mathrm{TE}_1},\ldots,\varphi_{\mathrm{TE}_\mathrm{I}}]^T, \\
\mathbf{g} &= [g_{\mathrm{TE}_1},\ldots,g_{\mathrm{TE}_\mathrm{I}}]^T.
\end{aligned} \tag{3}$$

The log-likelihood function of $s(n,\mathrm{TE})$ can be derived as

$$ln(L(s(n,\mathrm{TE});\boldsymbol{\theta})) = const - \frac{1}{\delta^2}\sum_{n=0}^{N-1}\|s(n,\mathrm{TE}) - \hat{s}(n,\mathrm{TE};\boldsymbol{\theta})\|^2, \tag{4}$$

where $L(s(n,\mathrm{TE});\boldsymbol{\theta})$ denotes the likelihood function (Gaussian). Then each block entry (denoted as F_{θ_1,θ_2}) of the overall FIM, $\mathbf{F}(\boldsymbol{\theta})$, can be calculated as:

$$F_{\theta_1,\theta_2} = \mathbb{E}\left[\left(\frac{\partial ln(L)}{\partial\theta_1}\right)\left(\frac{\partial ln(L)}{\partial\theta_2}\right)\right]. \tag{5}$$

The final FIM can be formed as

$$\mathbf{F}(\boldsymbol{\theta}) = \begin{bmatrix} F_{c,c} & F_{c,T_2} & F_{c,T_2'} & F_{c,\Delta f} & F_{c,\varphi} & F_{c,g} \\ F_{c,T_2}^H & F_{T_2,T_2} & F_{T_2,T_2'} & F_{T_2,\Delta f} & F_{T_2,\varphi} & F_{T_2,g} \\ F_{c,T_2'}^H & F_{T_2,T_2'}^H & F_{T_2',T_2'} & F_{T_2',\Delta f} & F_{T_2',\varphi} & F_{T_2',g} \\ F_{c,\Delta f}^H & F_{T_2,\Delta f} & F_{T_2',\Delta f} & F_{\Delta f,\Delta f} & F_{\Delta f,\varphi} & F_{\Delta f,g} \\ F_{c,\varphi}^H & F_{T_2,\varphi}^H & F_{T_2',\varphi}^H & F_{\Delta f,\varphi}^H & F_{\varphi,\varphi} & F_{\varphi,g} \\ F_{c,g}^H & F_{T_2,g}^H & F_{T_2',g}^H & F_{\Delta f,g}^H & F_{\varphi,g}^H & F_{g,g} \end{bmatrix}.$$

The lower triangle of $\mathbf{F}(\boldsymbol{\theta})$ is the conjugate transpose of the upper triangle part, thus the whole matrix can be generated after obtaining all the entries above the diagonal using Eq. (5). Detailed calculations for each entry can be found similarly in Refs. [1, 2]

References

[1] H. Nguyen, *Towards high-resolution magnetic resonance spectroscopic imaging: spatiotemporal denoising and echo-time selection.* University of Illinois at Urbana-Champaign, 2011.

[2] Q. Ning, *Spectral estimation with spatio-spectral constraints for magnetic resonance spectroscopic imaging.* University of Illinois at Urbana-Champaign, 2015.

Supplementary Figures

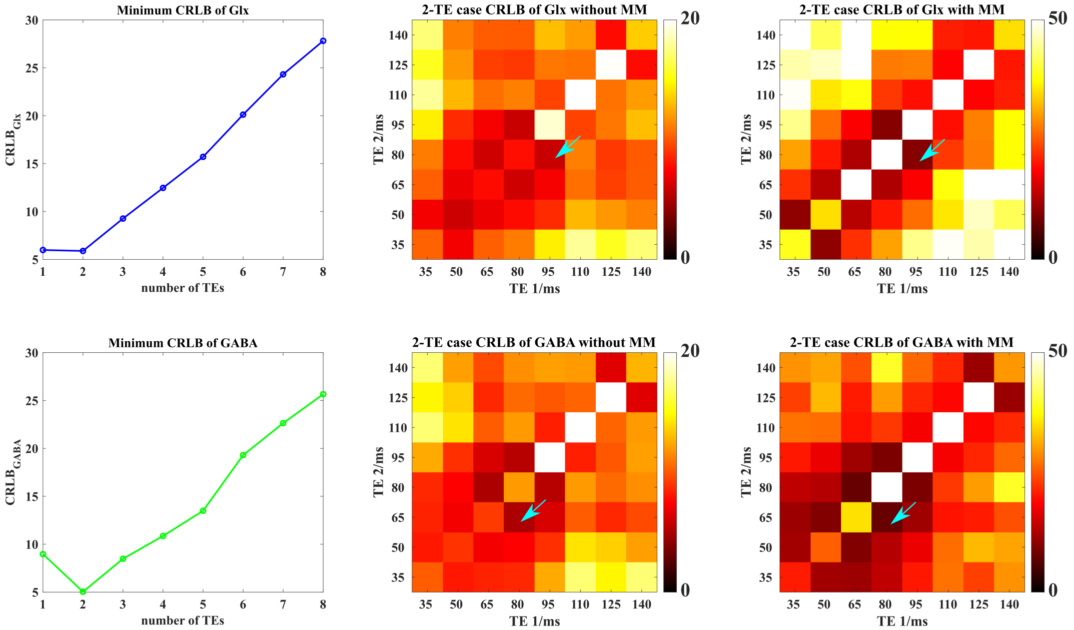

Figure S1: CRLB analysis and TE optimization for the estimation of Glx (Row 1) and GABA (Row 2). Column 1 shows the minimum CRLB w.r.t. the number of TEs identified using a greedy search (sequentially adding new TEs into the optimization) without considering macromolecule signals (without MM). Columns 2 and 3 show the CRLBs for all possible 2-TE combinations (in the range of 35 to 140 ms with 15 ms increment) without and with R1.1
MM signals, respectively. Note that the range of CRLBs without MM is [0, 20] in the figure, and the range of CRLBs with MM is [0, 50].
The 2-TE acquisition, in an equivalent time comparison, yielded the lowest CRLB for GABA while the difference between 1 and 2 TEs for Glx is small. Noting that the optimal 2-TE combinations for Glx and GABA (marked by the blue arrows) are different, which can be due to the inherent resonance structure and J-coupling differences between these two components. Considering that GABA estimation is more challenging, we chose the 2 TEs of 65 and 80 ms which minimize the GABA CRLB for in vivo acquisition.

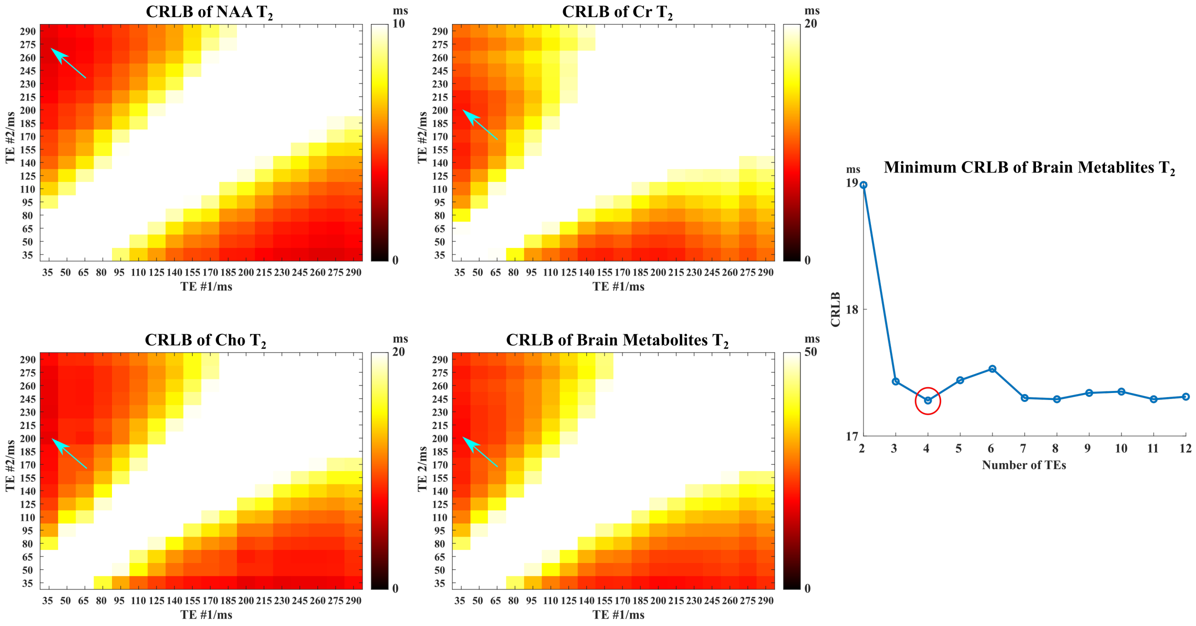

Figure S2: CRLB analysis and TE optimization for metabolite T_2 estimation. Columns 1 and 2 show the CRLB of NAA, Cr, Cho T_2's and their combination (in ms) w.r.t. 2-TE choices. The TE combinations with the lowest CRLB were marked by the blue arrows. The plot on the right shows the minimum CRLB at different numbers of TEs acquired (jointly considering NAA, Cr and Cho), under an equivalent time constraint (repeated TE selections allowed while adding new TEs). The globally minimal CRLB can be achieved with 4 TEs (i.e., 35, 200, 245, and 275 ms), which were used for in vivo data collection.

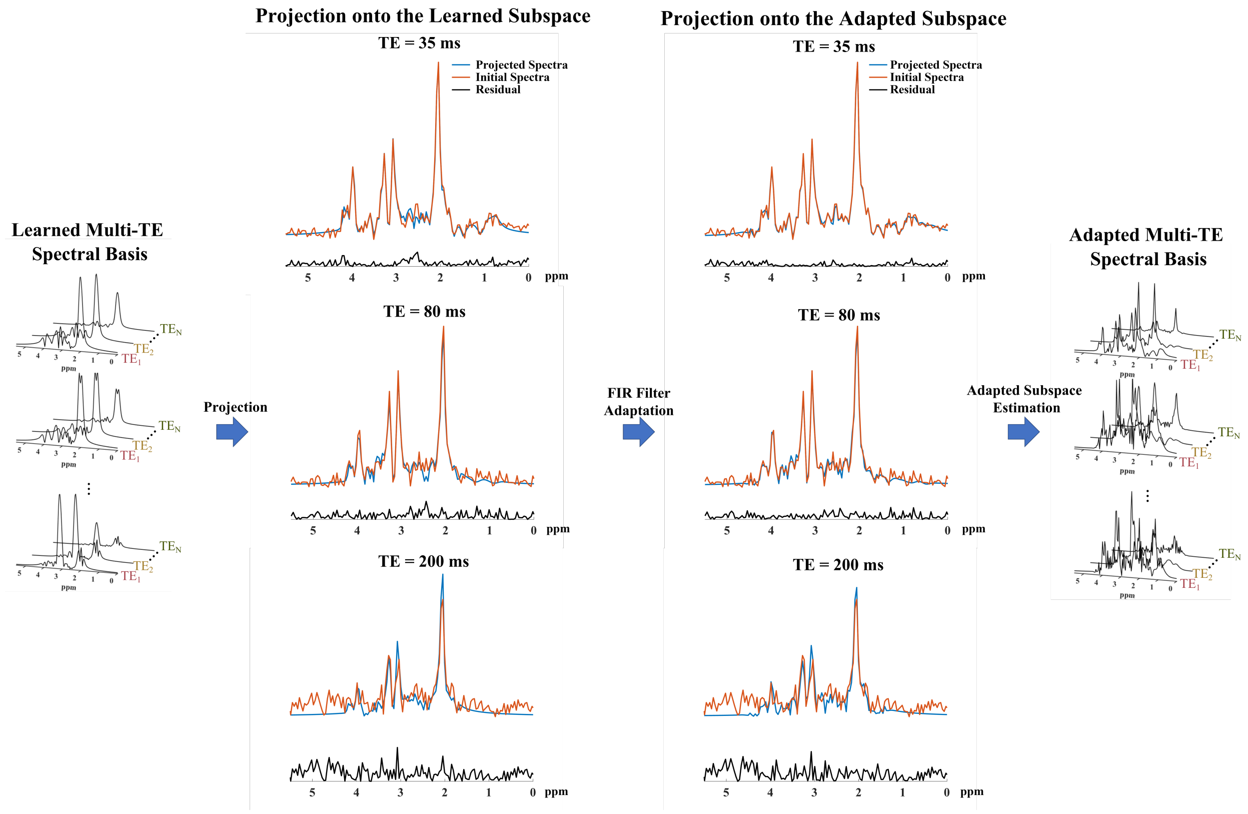

Figure S3: Subspace learning and adaptation: After obtaining the learned multi-TE subspace (shown in the form of spectral basis functions here) from the training data (first column), we projected high-SNR experimental data onto the learned subspace to get reference signals (second column). Then a voxel-wised FIR filter was estimated to adapt the reference signals to the actual experimental data (with additional lineshape variations). Smaller residuals can be observed after this adaptation (third column), indicating better representation capability. Finally, an adapted subspace (last column) can be generated from the refitted data.

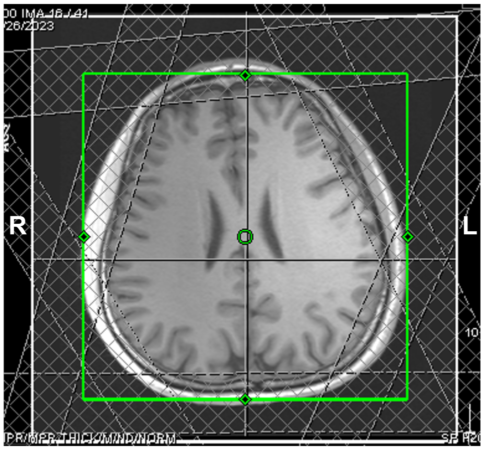

Figure S4: Experimental setup from a healthy volunteer scan. The shaded areas are the OVS bands for subcutaneous fat suppression (only 6 showing up in this in-plane view, the other 2 set at the top and bottom of the imaging volume). The green box is the shimming volume.

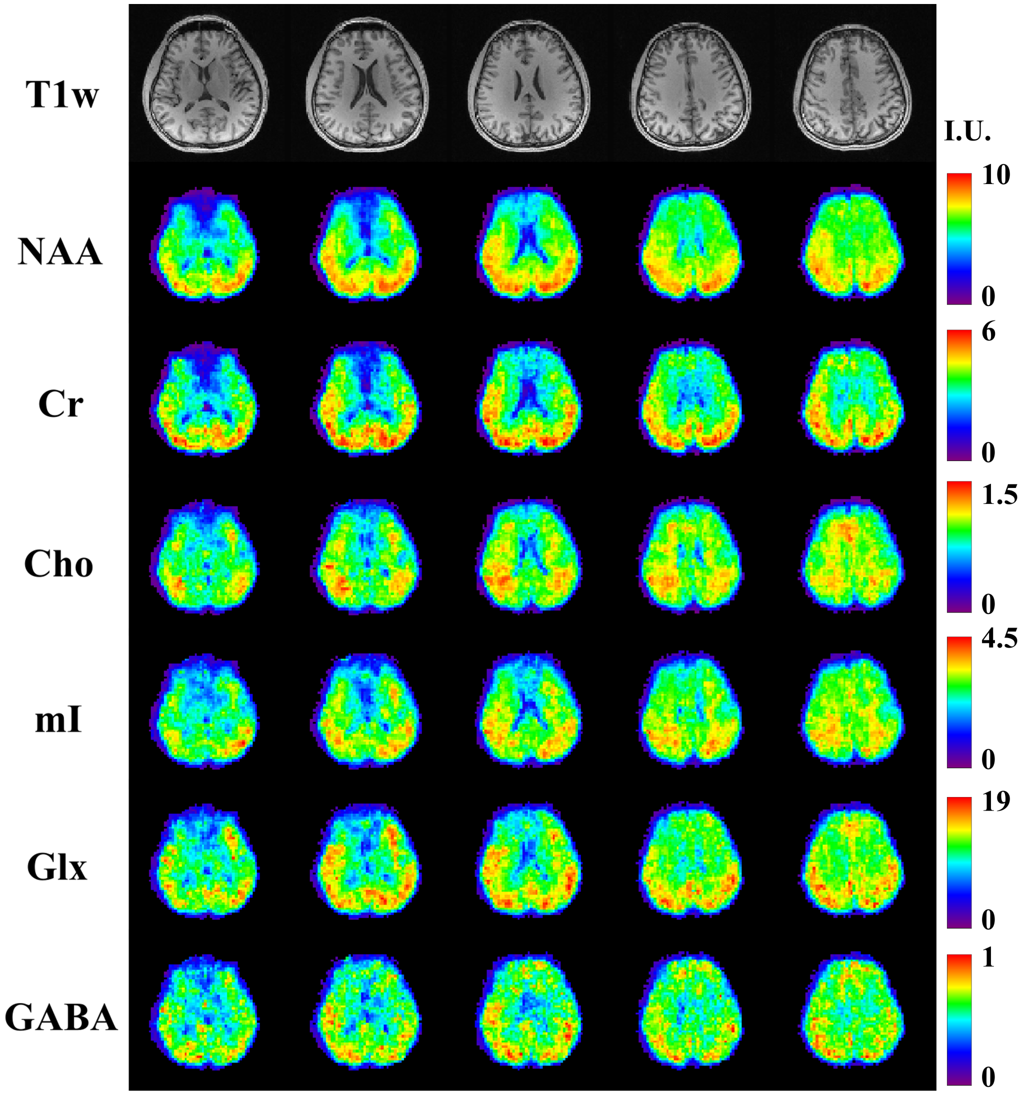

Figure S5: 3D metabolite and neurotransmitter mapping results from volunteer #2. The figure arrangement is the same as Fig. 7 in the main text.

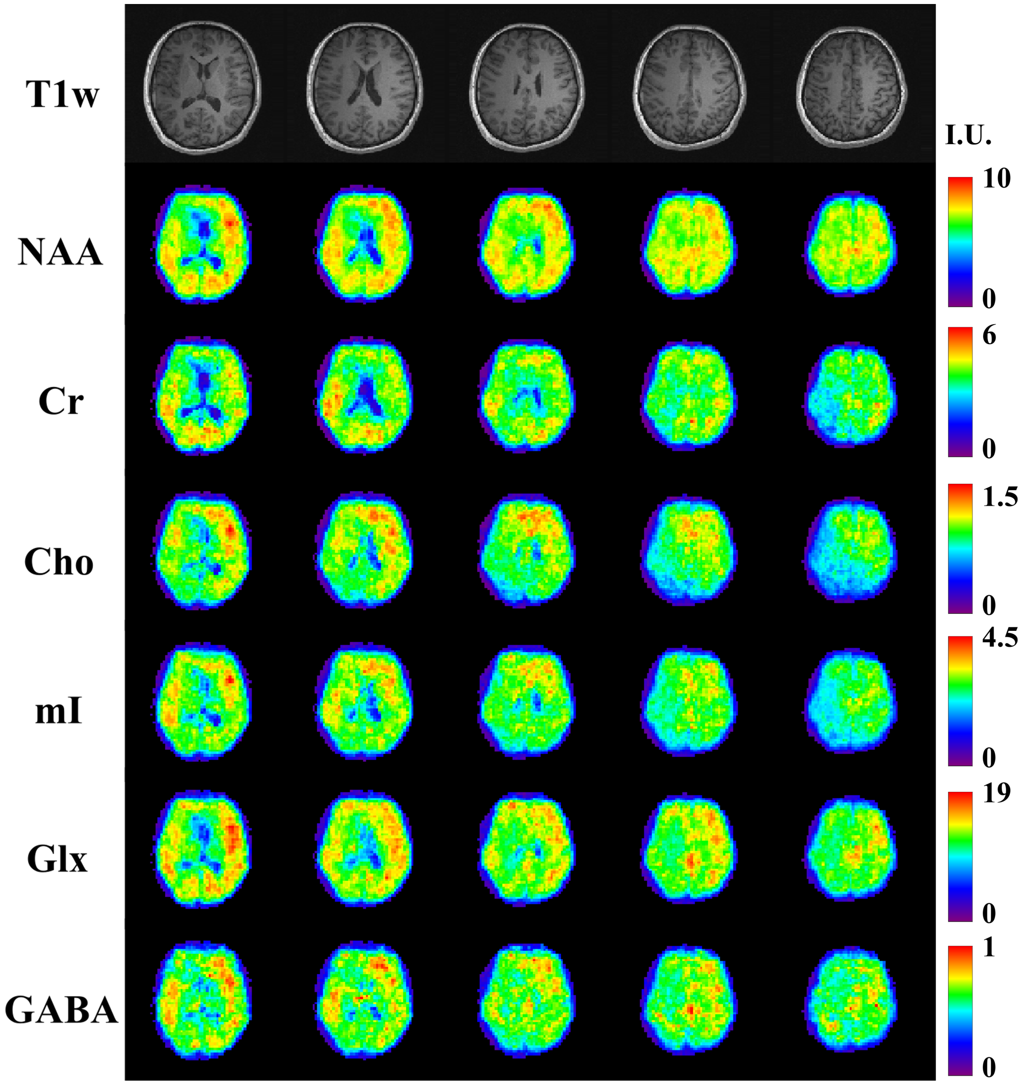

Figure S6: 3D metabolite and neurotransmitter mapping results from volunteer #3. The figure arrangement is the same as above.

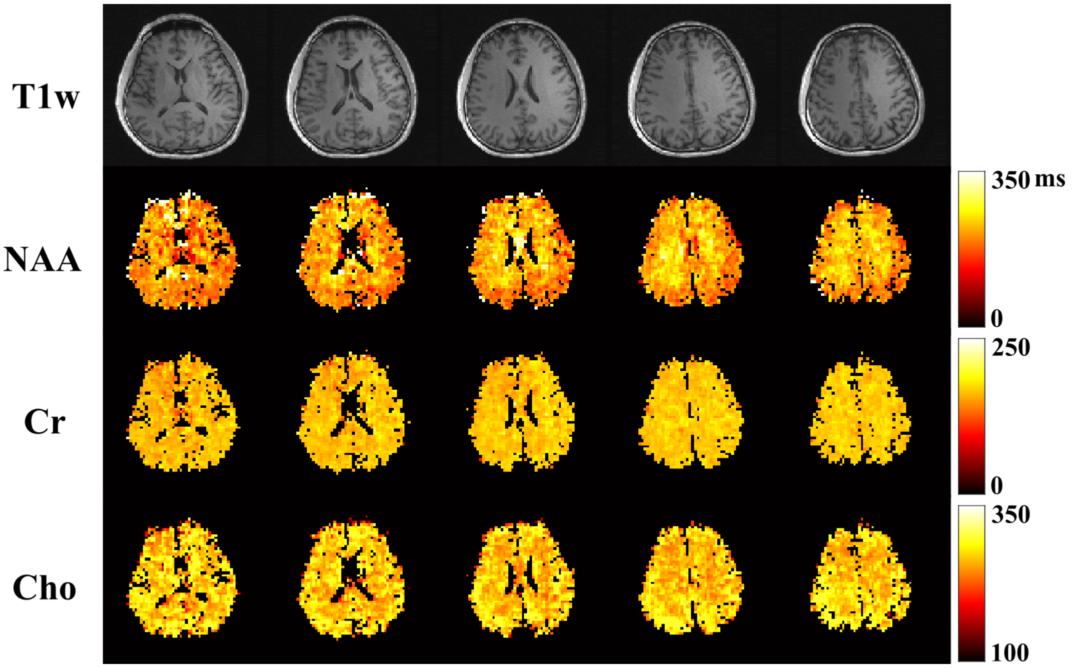

Figure S7: High-resolution 3D metabolite T_2 mapping results from volunteer 2. The figure arrangement is the same as Fig. 9 in the main text.

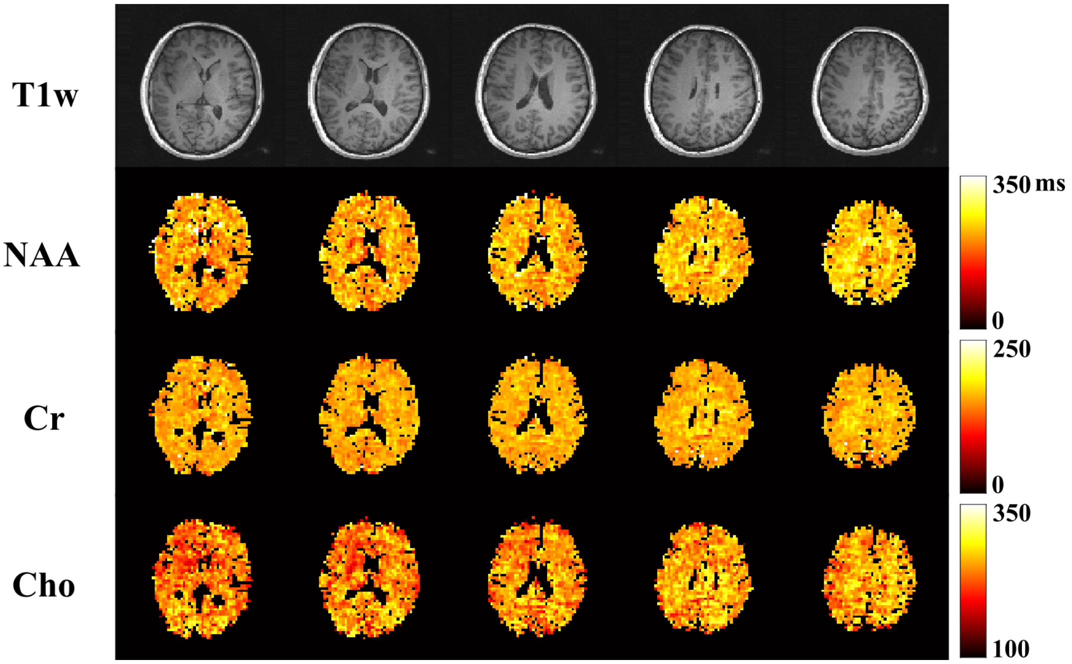

Figure S8: High-resolution 3D metabolite T_2 mapping results from volunteer 3. The figure arrangement is the same as above.

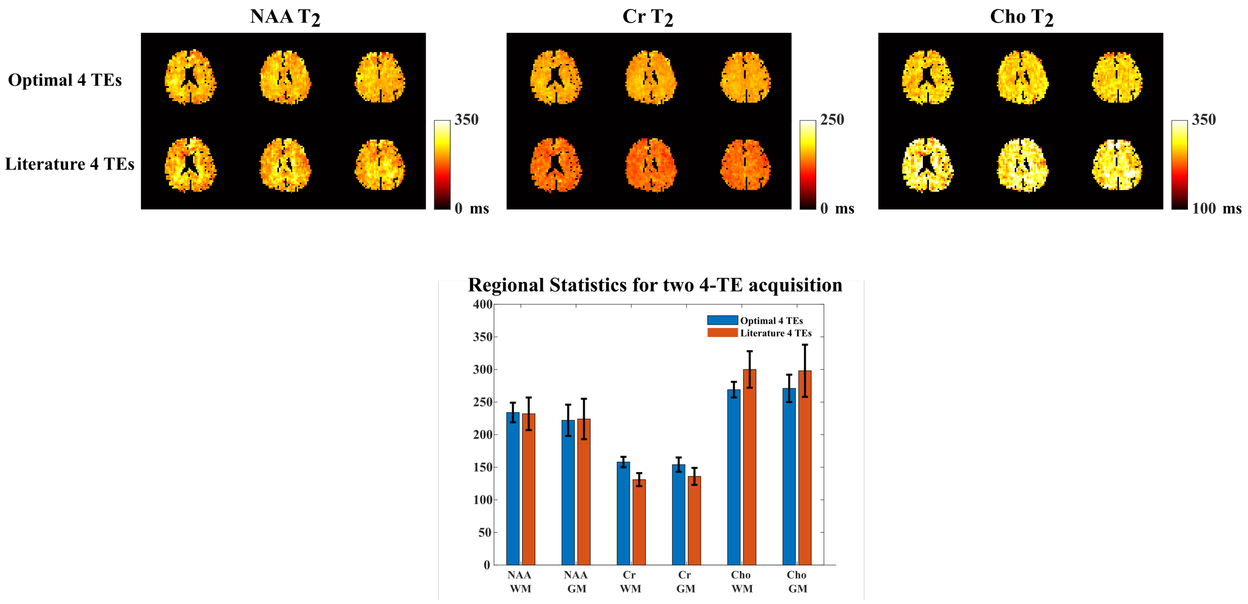

Figure S9: Comparison of optimal 4-TE (top row) and literature 4-TE (second row) acquisitions for metabolite T_2 mapping. Regional values are shown in the bottom panel. For the optimal 4-TE acquisition: NAA T_2 is 234±15 ms in WM and 222±24 ms in GM, Cr T_2 is 158±8 ms in WM and 154±11 ms in GM, Cho T_2 is 269±12 ms in WM and 271±21 ms in GM. For the literature 4 TEs: NAA T_2 is 232±25 ms in WM and 224±31 ms in GM, Cr T_2 is 131±10 ms in WM and 136±13 ms in GM, and Cho T_2 is 300±28 ms in WM and 298±40 ms in GM. All regional statistics were obtained within the same subject across the imaging slices shown in the figure. The optimal 4-TE acquisition reduced the variance and mitigated the underestimation and overestimation for Cr and Cho T_2's.

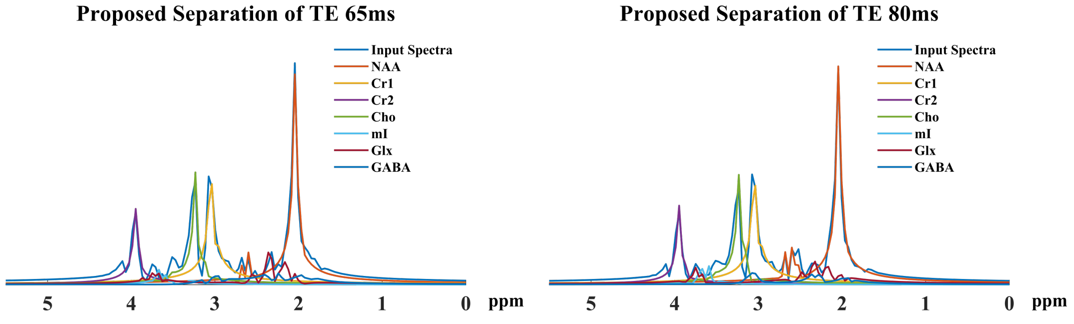

Figure S10: Separated spectral components for a representative 2-TE in vivo spectra: both the overall spectrum and the separated components are shown in the same plot for each TE.

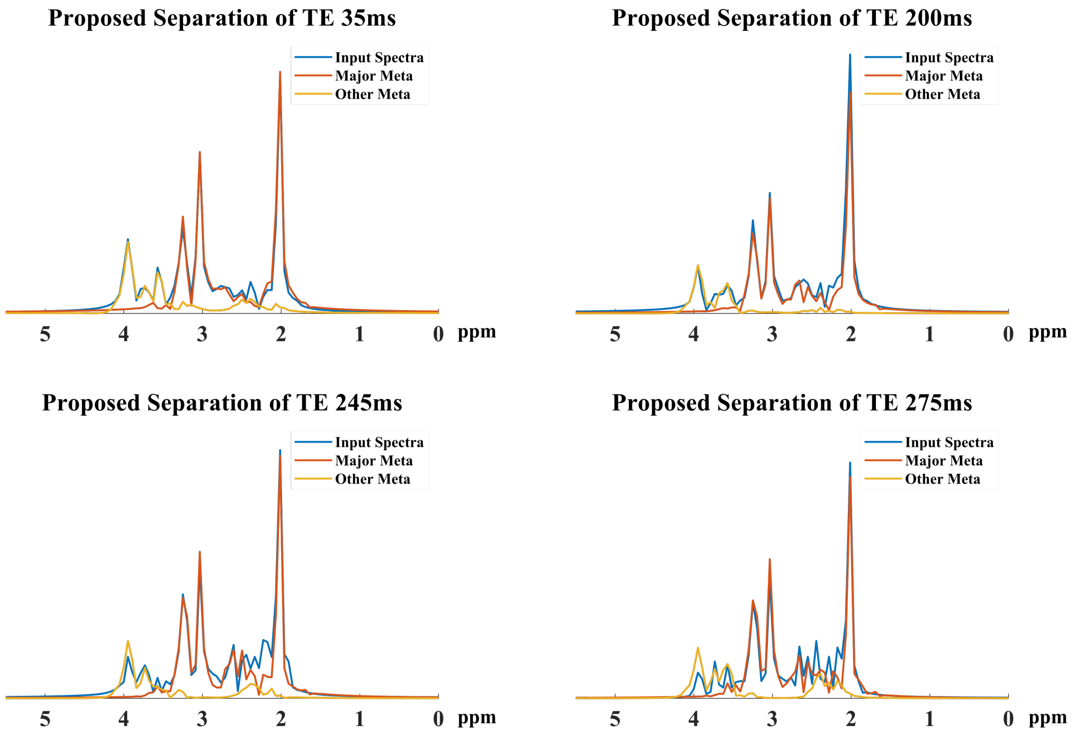

Figure S11: Separated spectral components for a representative 4-TE in vivo spectra.

Region	NAA	Cr	Cho
**White Matter**	258.51±46.33 ms	155.35±13.83 ms	265.29±21.39 ms
**Grey Matter**	226.55±49.90 ms	151.09±15.93 ms	259.46±36.90 ms

Table S1: Metabolite T_2 statistics (mean ± standard deviation) in grey and white matters computed from the same dataset (Fig. 9 in the main text) across different voxels. NAA T_2 in WM is significantly higher than GM, while Cr and Cho show nonsignificant difference between the two tissue types. The overall T_2 ranges for different metabolites are consistent with previously published results.

Region	NAA	Cr	Cho
**Frontal GM**	211.85±38.51 ms	156.95±21.47 ms	249.40±35.70 ms
**Temporal GM**	209.23±33.62 ms	157.08±17.95 ms	245.52±32.58 ms
**Occipital GM**	210.01±37.44 ms	154.61±21.83 ms	249.83±37.72 ms

Table S2: Regional metabolite T_2's (mean ± standard deviation) summarized from all the volunteers' data.
